# Pattern enrichment analysis for phage selection of stapled peptide ligands†

**DOI:** 10.1039/d2sc04058a

**Published:** 2022-10-12

**Authors:** Takayuki Miki, Keigo Namii, Kenta Seko, Shota Kakehi, Goshi Moro, Hisakazu Mihara

**Affiliations:** School of Life Science and Technology, Tokyo Institute of Technology 4259 Nagatsuta-cho, Midori-ku Yokohama Kanagawa 226-8501 Japan tmiki@bio.titech.ac.jp

## Abstract

Phage display is the most widely used technique to discover *de novo* peptides that bind to target proteins. However, it is associated with some challenges such as compositional bias. In this study, to overcome these difficulties, we devised a ‘pattern enrichment analysis.’ In this method, two samples (one obtained by affinity selection, the other simply amplified without selection) are prepared, and the two sequence datasets read on next-generation sequencer are compared to find the three-residue pattern most enriched in the selected sample. This allows us to compare two sequence datasets with high coverage and facilitates the identification of peptide sequences and the key residues for binding. We also demonstrated that this approach in the combination with structured peptide libraries allowed spatial mapping of the enriched sequence patterns. Here, we prepared a phage library displaying chemically stapled helical peptides with the X_1_C_2_X_3_X_4_X_5_X_6_X_7_X_8_C_9_X_10_ sequence, where X is any amino acid. To validate our method, we performed screening against the HDM2 protein. The results showed that the hydrophobic residues (Phe, Tyr, Trp and Leu) that are key to interactions with HDM2 were clearly identified by the pattern enrichment analysis. We also performed selection targeting the SARS-CoV-2 spike RBD in the same manner. The results showed that similar patterns were enriched among the hit peptides that inhibited the protein–protein interaction.

## Abstract 2

To effectively identify helical peptide binders from a phage library, we developed “pattern enrichment analysis,” which finds the enriched three-residue patterns by comparison with a control sample.

## Graphical abstract

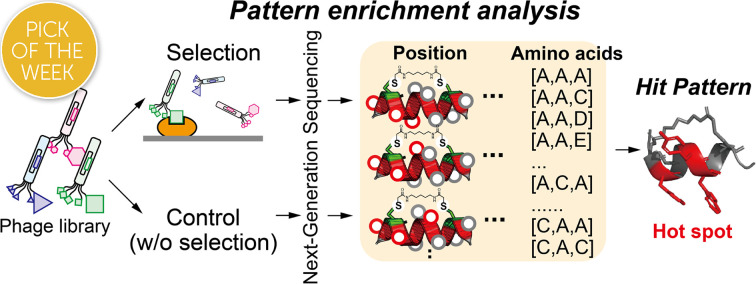

## Introduction

Peptide-based inhibitors are promising therapeutic modalities.^1^ Owing to their large contact surfaces, peptides have the potential to inhibit protein–protein interactions and exhibit high specificity to target molecules, which is difficult to achieve with small molecules.^2^ Another significant advantage is the accessibility of organic synthesis. Introducing noncanonical amino acids, chemical modifications and cyclisation can convert suboptimal lead peptides into high-affinity inhibitors with high proteolytic stability.^1,3^

Traditionally, most peptide drugs have been developed based on natural sequences.^4^ With the development of *in vitro* evolution using genetically encoded libraries such as phage,^5,6^ yeast^7^ and mRNA^8,9^ display, intensive efforts have been made to discover bioactive *de novo* peptides. Among these options, phage display is a system applied worldwide, and commercially available phage libraries such as the Ph.D.-C7C and Ph.D.-12 libraries are widely used. Despite its versatility, several key challenges are associated with phage display. First, complicated techniques need to be mastered to efficiently obtain ligands with high affinity. Given that some background noise will inevitably develop due to nonspecific interactions, the selection stringency must be appropriately adjusted to differentiate the desired phages from nonspecific phages.^6^ Considering the phage recovery yield in each round, it is necessary to tune various parameters such as the amount of epitope, the concentration of phage, the number of washes and the buffer contents. In addition, phage display suffers from various biases.^10^ The ‘NNK’ codon (N is A, T, G or C; K is T or G) is commonly incorporated at a random position, although there is compositional bias due to the codon redundancy. Moreover, the infection and amplification rates of phages depend on the presented peptide sequence.^11^ During the consecutive repeated processes of selection and amplification, rapidly growing clones named ‘parasitic clones’ are readily enriched. According to a report by Derda's group, the deep sequencing of amplified phages without binding selection identified 770 parasitic clones in the Ph.D.-7 library, 197 of which were sequences identified in the literature.^12^ Hence, the amplification bias imposes inefficient selection.

Next-generation sequencing (NGS) or deep sequencing have been reported to reduce the effects of such bias, where enriched sequences have been identified by comparing phage pools without binding selection (control experiment).^13,14^ However, when the diversity of the phage library is enormous (theoretically 20^8^ peptides when eight residues are randomised), the number of NGS reads (10^5–6^) is orders of magnitude less than the diversity in the library and cannot cover even 1% of the total. Thus, when comparing two different sequence subsets, most of the sequences are detected in either one or the other, restricting the usage of enrichment analysis.

**Fig. 1:**
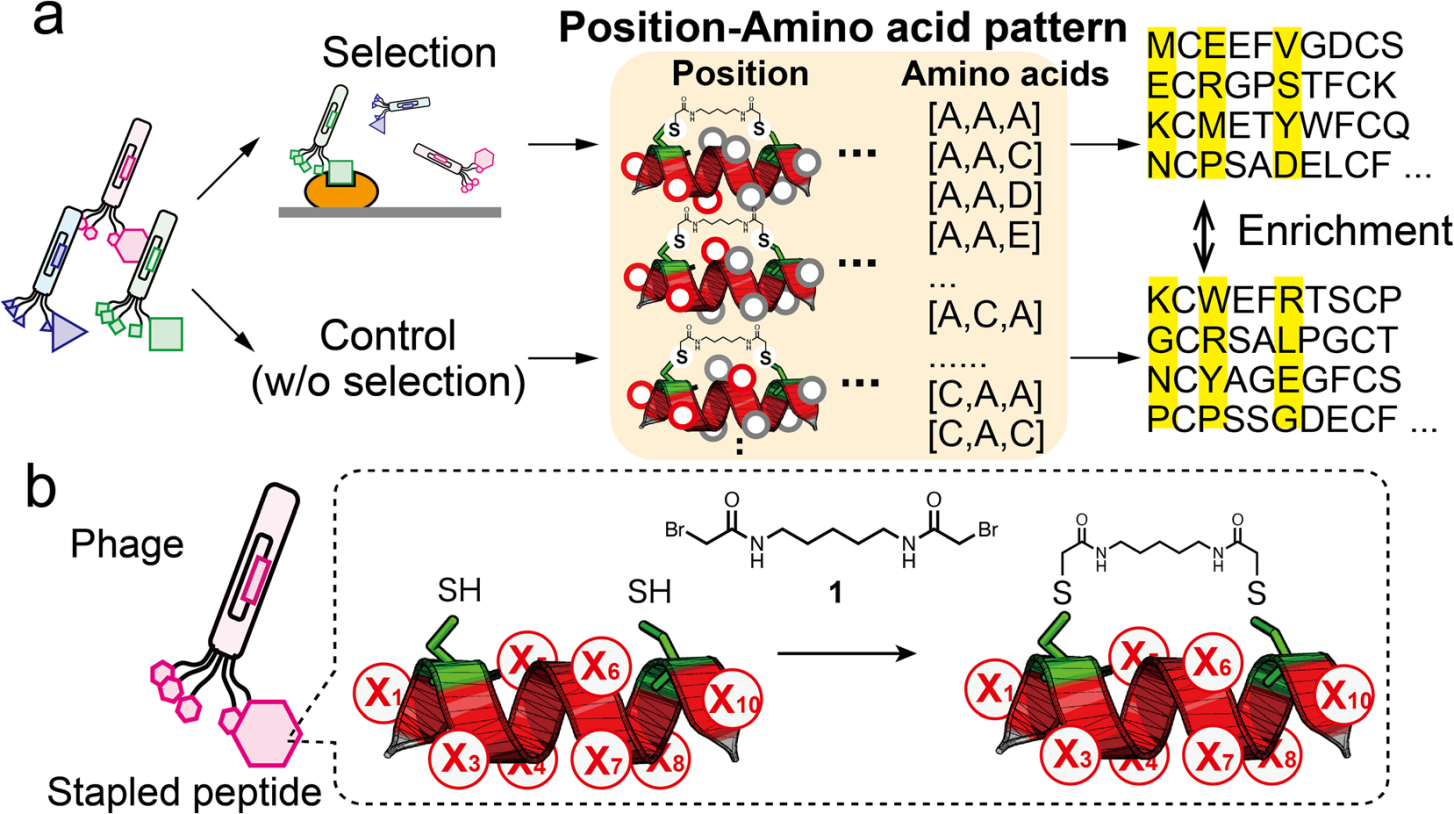
Strategy of pattern enrichment analysis for peptide ligand selection. (a) Schematic illustration of the bio-panning and the pattern enrichment analysis. In the analysis, for each of the three-residue position (red circles) patterns, enrichment values of all amino acid patterns were calculated by comparing the sequence dataset of the selected samples with that of the control. (b) Design of randomised peptide library (X, random residue) and the modification reaction with compound 1.

In this report, we demonstrate ‘pattern enrichment analysis’, which comprehensively calculates enrichment by focusing on all three-residue positions (Fig. 1a). This concept is based on fundamental insights into natural protein–protein interactions, involving ‘hotspots’ consisting of a few key residues.^15,16^ For example, three hydrophobic residues Phe9, Pro10 and Pro13 of MBM1 (menin binding motif 1) at its interaction site are critical for binding to menin.^17^ Because the discovery of hotspots is of primary importance in phage display, we envisage that pattern enrichment analysis focusing on a few residues is productive. Moreover, restricting the analysis to three positions can reduce the theoretical sequence patterns to 8000 (=20^3^), which is a smaller number than for NGS reads. When NGS reads 10^5^ sequences, each single pattern is counted 12.5 times on average, facilitating quantitative evaluation between two subsets. Comparison with a control (without binding selection) would reduce the compositional bias and lead to the practical identification of hotspots.

We also hypothesise that the combination of the above-mentioned approach with a structured peptide library would be effective because the sequence patterns obtained by the analysis have spatial implications. Mapping these residues on the peptide scaffold would reveal the spatial sites critical for binding to the target. Here, to obtain helical peptide ligands, we prepared a phage library displaying chemically stapled peptides containing eight randomised residues. For validation of our strategy, three rounds of screening from the library were carried out against HDM2, and the sequence datasets were read by NGS analysis. The pattern enrichment analysis successfully identified the hotspot residues for HDM2 binding. Furthermore, we performed the selection against SARS-CoV-2 spike RBD. The identified peptides exhibited the potential to inhibit the interaction between SARS-CoV-2 spike RBD and ACE2.

## Result & discussion

### Design of a phage library displaying stapled peptides

Helical structures are frequently present at interfaces where natural protein–protein interactions occur.^18^ Hence, a helical peptide is a great backbone candidate for ligand selection. A practical method for stabilising the α-helical conformation is to use stapling linkers that connect two residues at *i*, *i* + 4 or *i*, *i* + 7 spacing on one face of the helix.^19^ The chemical stapling improves the binding affinity by reducing the entropic penalty and enhances the resistance to proteolysis. Various helical peptides stapled by ruthenium-catalysed olefin metathesis have been exploited.^20^ The stapling approach has been adopted in phage display. The development of disulphide-free gene-3-protein facilitates selective chemical modification of fd phage to introduced nucleophilic cysteines without impairing phage function.^21,22^ Heinis' group constructed a variety of bicyclic peptide phage libraries^23,24^ and also a stapled peptide library with the *i*, *i* + 4 position.^25^ Our group has reported stapled libraries with the *i*, *i* + 7 position to select helical peptide ligands.^26,27^

In this study, we designed a phage library displaying the XC6CX (X_1_C_2_X_3_X_4_X_5_X_6_X_7_X_8_C_9_X_10_, C: cysteine, X: random residue) peptide with two cysteine residues at *i* and *i* + 7 (Fig. 1b). For library construction, the ‘NNK’ codon was introduced at each of eight randomised residues (Fig. S1†). A phage library presenting stapled peptides was constructed by chemical modification with a cross-linker (1) containing two bromoacetyl groups.^26^

### Initial and amplification bias of the peptide library

**Fig. 2:**
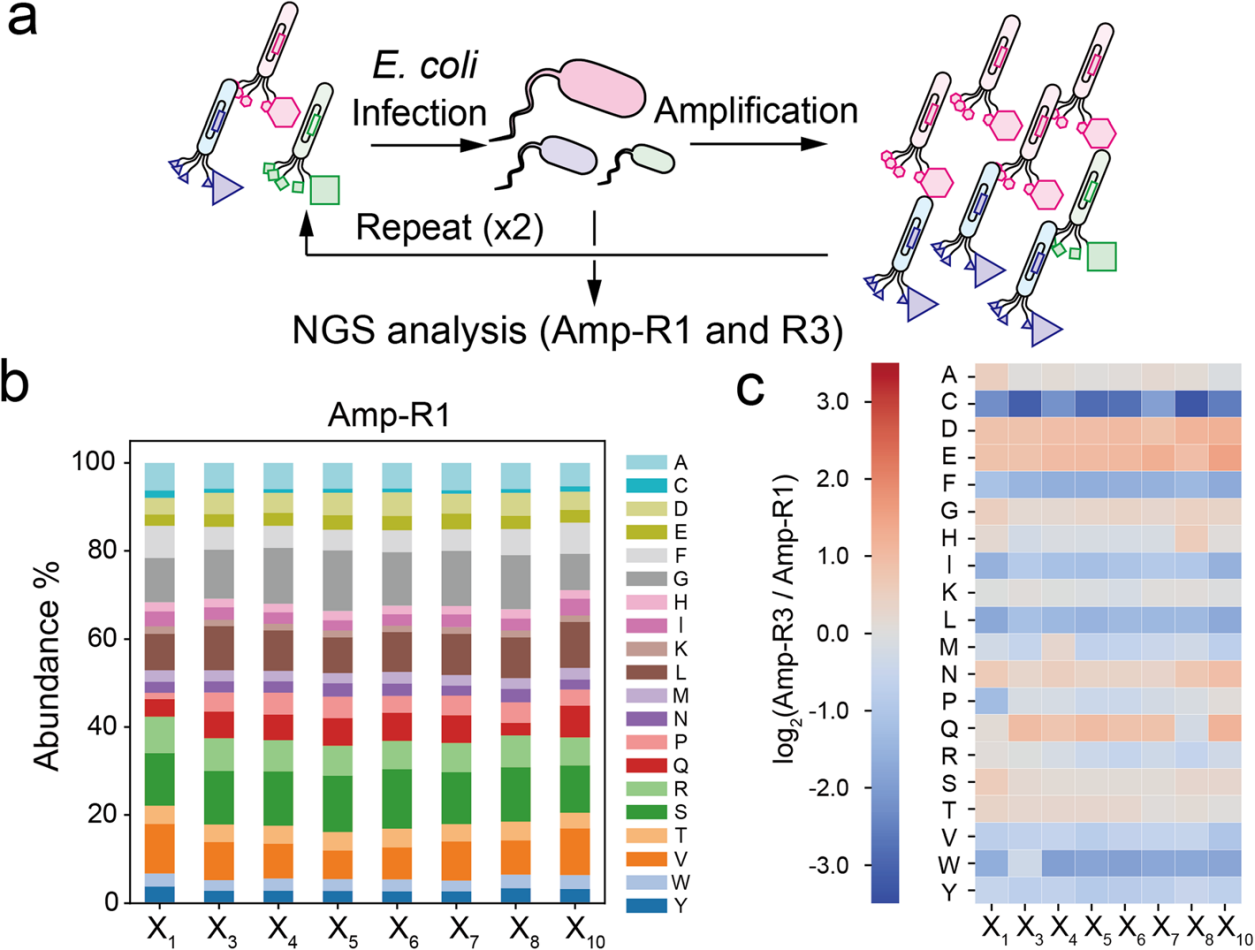
Compositional bias in the phage library. (a) Preparation of simply amplified phage libraries (Amp-R1 or Amp-R3: single-cycle or three-cycle amplified phage pool, respectively). (b) Amino acid composition at each position in the Amp-R1 pool. (c) Heatmap of log_2_(ratio) value of Amp-R3 *versus* Amp-R1.

To investigate the compositional bias of the phage library at the initial and amplified stages, we prepared two *E. coli* TG-1 samples infected by the naïve XC6CX phage library (Amp-R1) or the phage pool after three consecutive cycles of infection and amplification (Amp-R3) (Fig. 2a). The phage vectors were extracted and subjected to NGS analysis to obtain over 300 000 reads for each sample (Table S1†). Data processing from the FASTQ format was performed based on Heinis' and Derda's reports^28,29^ (Fig. S2†). Amp-R1 has a large diversity of sequences (371 325 unique sequences among 389 893 reads), and 91% of which are present as a single copy. While the nucleotides in the ‘NNK’ sequence were almost identically introduced in Amp-R1 as expected (Fig. S3†), the amino acid frequencies were not equal mainly due to codon redundancy (Fig. 2b). Thus, the ‘NNK’ degenerate codons are inexpensive yet strongly biased in their initial composition. The amplification cycle attenuated the sequence diversity, leading to 66% of total reads in Amp-R3 being present as a single copy (Fig. S4†). The initial bias remained even in Amp-R3 (Fig. S5†). Comparing the amino acid frequencies at each position of Amp-R3 with those of Amp-R1 showed an overall increase in hydrophilic amino acids (D, E, N, Q) and a decrease in hydrophobic ones (F, I, L, V, W, Y) and cysteine, indicating significant amplification bias (Fig. 2c). Two further rounds of amplification (Amp-R5) resulted in a dramatic reduction in peptide diversity and pronounced compositional bias (Fig. S4 and S5†).

### Selection of peptide ligands against HDM2

As a proof-of-concept of our pattern enrichment analysis, we performed phage selection of peptide ligands against the HDM2 (human double minute 2) protein. HDM2 binds directly to the N-terminal helical peptide of p53, inhibiting p53-dependent transcription and inducing p53's proteasomal degradation.^30^ The hotspots of the interaction are well defined as three hydrophobic residues (Phe19, Trp23 and Leu26) on the one side of the p53 helical peptide directly contacting the HDM2 pocket.^31,32^ Various stapled peptide inhibitors were optimised based on the triad sequence.^33–35^ In the studies against the HDM2 protein, we investigated how our strategy reduces the bias and facilitates the identification of hotspots.

**Fig. 3:**
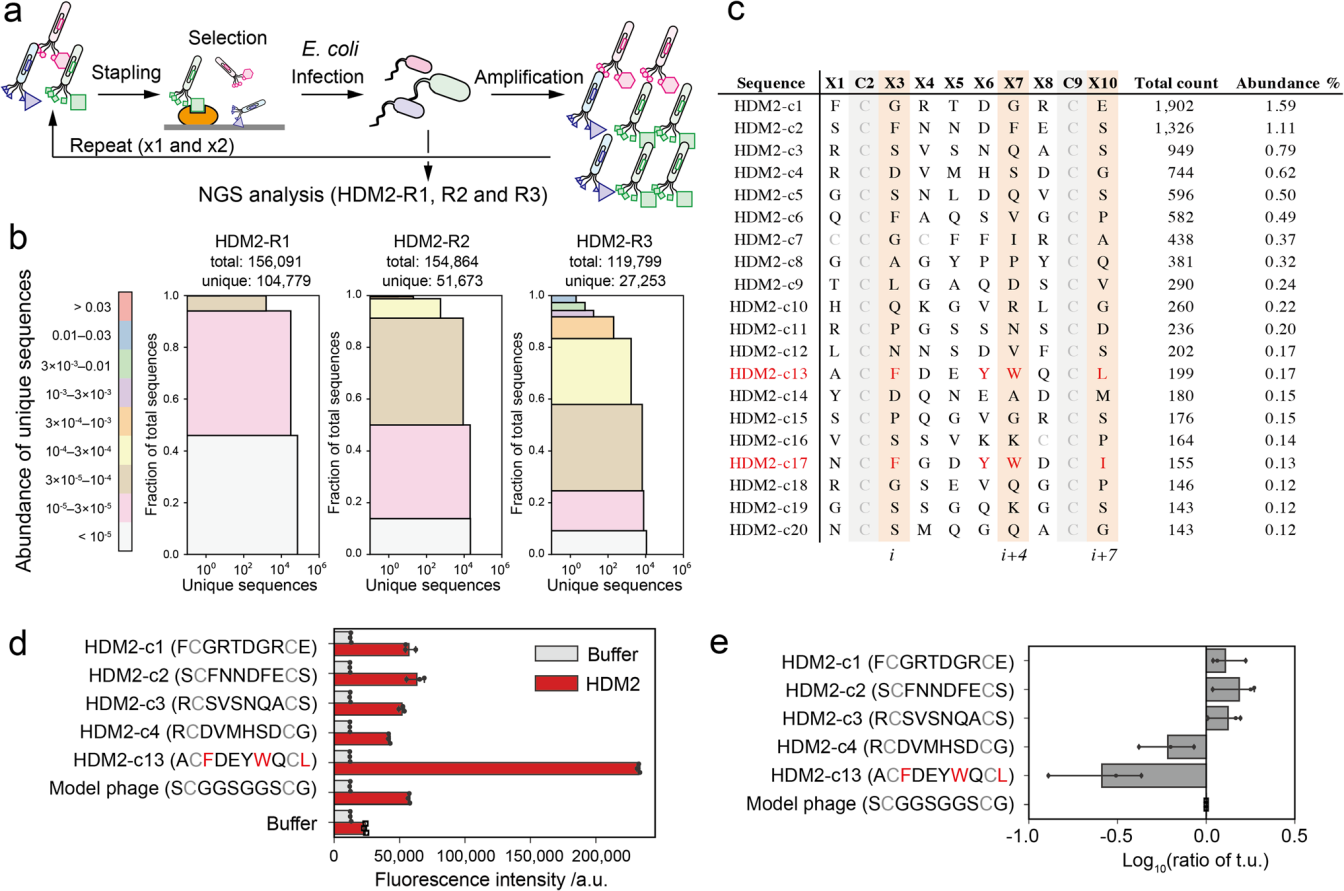
Phage selection of HDM2-binding peptide ligands. (a) Screening scheme of the stapled peptide tethered phage library (HDM2-R1, R2 or R3: selected phage pools after single, double or triple binding selections, respectively). (b) Diversity plots of phage pools after selection. All unique sequences obtained by NGS analysis were grouped by their abundance (coloured segments). For instance, the grey segment (<10^5^) contains unique sequences counted only a single time. For each coloured segment, the number of unique sequences and the proportion of total reads are shown on the *x*- and *y*-axes, respectively. (c) The top 20 sequences in HDM2-R3 pools. Peptides (c13 and c17) containing F, W and L/I at *i*, *i* + 4 and *i* + 7 positions are coloured red. (d) Phage ELISA results against immobilised HDM2. Error bar represents SD (*n* = 3). (e) Relative infectivity of phages. The titres of each phage were normalised by that of model phage containing the S̲CG̲G̲S̲G̲G̲S̲CS̲ sequence. Error bar represents SD (*n* = 3).

The phages were quantitively modified by reagent 1 without impairing the phage activity (Fig. S6†). Then, the phage library displaying stapled peptides was incubated in an HDM2-immobilised plate. After washing unbound phages, bound phages were eluted in acidic buffer (pH 2.2, 50 mM Gly-HCl), infected into TG-1 cells and amplified. We performed three rounds of this selection (HDM2-R1, R2 and R3) (Fig. 3a and S7†). These samples were then subjected to NGS analysis to obtain over 100 000 reads (Table S2†). NGS analysis revealed that peptide diversity gradually decreased with the number of rounds of panning (Fig. 3b). Even after three rounds, only two of the clones reached abundance of 1% (Fig. 3c). Among the clones in the top 20, only a single clone ranked 13th (HDM2-c13) contained F, W and L at positions *i*, *i* + 4 and *i* + 7 (Fig. 3c). For the top four clones (HDM2-c1, 2, 3 and 4) and an FWL-containing clone (HDM2-c13), the binding to HDM2 was evaluated by ELISA. All but HDM2-c13 did not significantly bind to HDM2, indicating that the top four clones were false positives (Fig. 3d). We noted that immobilisation of HDM2 caused an increase in the background signal for all phages, indicating nonspecific interaction. From phage titre tests, HDM2-c1, c2 and c3 showed higher infectivity than the control phage containing a GGS sequence at random positions, while HDM2-c13 showed predominantly low infectivity (Fig. 3e). Taking these findings together, both nonspecific adsorption to HDM2 and amplification bias were assumed to have caused the false-positivity of the top clones. It is therefore considerably more difficult to identify peptide ligands for HDM2 based on count values.

Previously other groups have successfully found HDM2-binding peptides from phage libraries. The main reasons for the difference should be the selection conditions. In some studies, phages bound to the immobilised HDM2 were eluted by adding a p53-peptide competitive binder at the order of mM concentration to obtain the peptides interacting with the p53-binding site.^36,37^ On the other hand, we eluted the bound phages under acidic conditions, and the acid elution inevitably makes it difficult to obtain the FWL-containing peptides. Chen's group has developed HDM2-binding peptides with acid elution.^38^ In this report, they performed four cycles of binding selection against HDM2 immobilised on beads, where only four out of the ten phages displayed FWL-containing peptides, and the remaining six eluted phages were inactive in ELISA. Although the result is much better than ours (only two out of twenty peptides were FWL-related; three cycles of selection against HDM2 immobilised on the plate), this also suggests the difficulty of conventional selection methods.

We compared all sequences in HDM2-R3 to those in Amp-R3. However, only 9.7% of HDM2-R3 sequences were detected in Amp-R3, and the majority of such proportions could not be calculated (Fig. S8†). When a value of 0.8 was substituted for these zero-count sequences to analyse the enrichment values, HDM2-c1, c2 and c4 sequences in the top counts were ranked lower in the whole sequence enrichment analysis, and HDM2-c13 emerged in fourth. However, we could not find any consensus sequence. The simple comparison is suitable for removing specific ‘parasite’ sequences but cannot account for the overall bias gradient. We also conducted a motif search in XSTREME, but no clear consensus sequences were obtained (Fig. S9†).

### Three-window enrichment analysis of phage pools after selection against HDM2

**Fig. 4:**
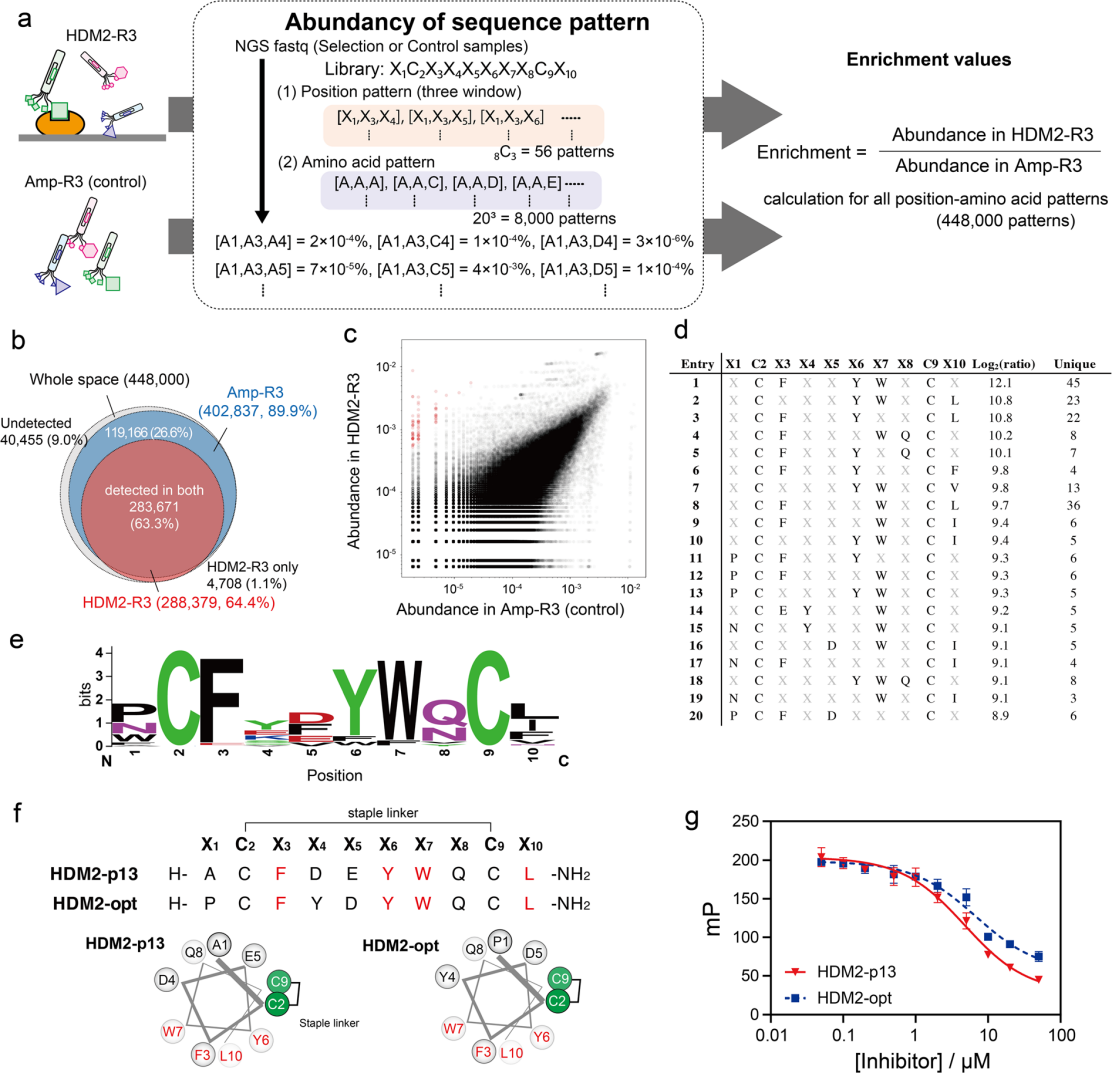
Position–amino acid pattern enrichment analysis of the HDM2-R3 pool. (a) Pattern enrichment score calculation. For both sequence data (FASTQ), the abundance of each position–amino acid pattern was determined. Enrichment values were calculated as the ratio of abundance in the selected sample to that in the control sample. (b) Venn diagram showing pattern coverage (white, whole space of three-window patterns; blue, patterns found in Amp-R3 dataset; red, patterns found in HDM2-R3 dataset). (c) Scattering plot indicating the abundance of control (*x*-axis) and HDM2-R3 selected sample (*y*-axis). Red circles are patterns with log_2_(ratio) values greater than 8. (d) Position–amino acid patterns ranked in the top 20. (e) WebLogo depicting enriched sequences determined from enriched patterns with log_2_(ratio) values greater than 8. (f) Sequences and helical wheel diagrams of HDM2-p13 and HDM2-opt peptides. Highly enriched residues are coloured red. (g) Fluorescence polarisation assay for competitive inhibition. Peptides (50 nM to 50 μM) were mixed with 100 nM HDM2 and 10 nM FAM-ATSP-3848. IC_50_ was calculated as 4.8 ± 0.6 and 6.9 ± 1.9 μM for HDM2-p13 (red) and HDM2-opt (blue), respectively. Error bar represents SD (*n* = 3).

We applied the above-mentioned datasets to pattern enrichment analysis *via* the following three steps (Fig. 4a). First, sequences at three arbitrary positions in the dataset were extracted. Next, for all amino acid patterns (20^3^ = 8000 amino acid patterns), the abundance was calculated; this calculation assigned a value of 0.8 counts for patterns with a zero count. The steps were repeated for all three-window positions (_8_C_3_ = 56 position patterns) to cover all position–amino acid patterns in both HDM2-R3 and Amp-R3 datasets. Finally, the enrichment value for each pattern was obtained as the abundancy ratio (HDM2-R3 *versus* Amp-R3). In this case, eight positions were randomised: 448 000 position–amino acid patterns (56 position patterns multiplied by 8000 amino acid patterns) in the three-window analysis. The results showed that the coverage of the Amp-R3 phage pool reached 89.9% (402 837 patterns) of all theoretical position–amino acid patterns (Fig. 4b). The pattern diversity of HDM2-R3 decreased with 64.4% coverage (288 379 patterns). Of these, 98.4% were also found in Amp-R3, facilitating quantitative evaluation. Fig. 4c shows the abundance of each pattern in the plots. The broad abundance distribution in Amp-R3 also supports the compositional bias (Fig. S10†). The abundance of patterns in Amp-R3 and HDM2-R3 showed a moderate positive correlation (*r* = 0.60), clearly indicating the presence of strong bias.

We focused on patterns with high enrichment [log_2_(ratio) > 8] (Fig. 4d). Here, patterns containing a Cys at any X position were excluded because they are inconsistent with the helical design. As such, most patterns contained F, Y and W at X_3_, X_6_ and X_7_ positions. In addition to these three residues, WebLogo plots showed an abundance of Pro or Asn at X_1_, acidic Asp and Glu at X_5_, Gln or Asn with an amide side chain at X_8_, and the hydrophobic Leu, Ile and Phe at X_10_ (Fig. 4e). These sequences agreed with the known stapled peptide ligands,^32^ suggesting that this method can precisely identify interaction hotspots (Fig. S11†). Interestingly, the crosslinking position was distinct from the reported hydrocarbon-stapled peptides. The hydrocarbon linker introduced between the residues after Phe and after Leu flanks the hydrophobic core.^34^ In contrast, the peptide enriched in this study was crosslinked between the Cys residues before Phe and before Leu, corresponding to the solvent-exposed face (Fig. 4f and S12†). Differences in stapling linker are likely to result in a discrepancy in stapling position. Nonetheless, the synthetic HDM2-p13 peptide exhibited helical content of 43% in 25% TFE buffer (Fig. S13†) and inhibited the high-affinity binding of fluorescently labelled ATSP-3848 (FAM-ATSP-3848) with HDM2 (ref. 33) (Fig. 4g and S14†). The stapled HDM2-opt peptide (P̲CF̲Y̲D̲Y̲W̲Q̲CL̲), whose sequence was determined from the pattern enrichment analysis, also exhibited effective inhibition (Fig. 4g and S15†).

### Phage selection of peptide ligands against SARS-CoV-2 spike RBD

**Fig. 5:**
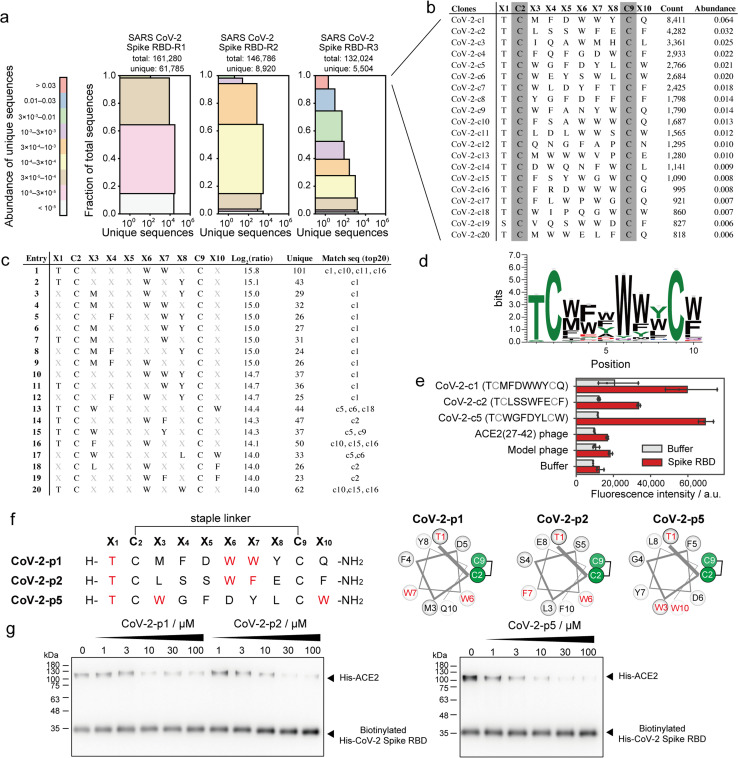
Phage selection of stapled peptide ligands against CoV-2 spike RBD. (a) Diversity plots of phage pools after selection against SARS-CoV-2 spike RBD. (b) The top 20 sequences in SARS-CoV-2 spike RBD-R3 pools. (c) The top 20 position–amino acid patterns enriched in the selected sample. (d) WebLogo depicting enriched sequences determined from pattern enrichment analysis with log_2_(ratio) values greater than 10. (e) Phage ELISA against immobilised CoV-2 spike RBD. Error bar represents SD (*n* = 3). (f) Sequences and helical wheel diagrams of CoV-2-p1, p2 and p5 peptides. The most enriched patterns are highlighted in red. (g) Pull-down assay for the inhibition of ACE2 and CoV-2 spike RBD interaction. IC_50_ was calculated as 7.8 ± 8.2, 12.9 ± 4.9 and 3.8 ± 3.3 μM for CoV-2-p1, p2 and p5, respectively (*n* = 3).

SARS-CoV-2 recognises ACE2 as the receptor by which it can enter cells.^39^ Various peptide binders for SARS-CoV-2 spike proteins have been developed using library screening techniques, such as affinity selection-mass spectrometry^40^ and mRNA display.^41^ To develop peptide inhibitors, we conducted selection of stapled peptide ligand against SARS-CoV-2 spike RBD in the same manner as the ligand selection against HDM2 (Fig. S16†). Compared with that upon selection against HDM2, peptide diversity was dramatically decreased. From NGS analysis, only 5504 unique peptides of 132 024 reads were detected after three rounds of selection (Fig. 5a). All top sequences contained Thr or Ser at X_1_, while the other positions were predominantly aromatic residues (Fig. 5b). The 11 peptides exceeded 1% in abundance, suggesting that the selection pressure was sufficiently stringent. The position–amino acid pattern matching the top sequences also ranked high in the pattern enrichment analysis (Fig. 5c). In particular, the top pattern (Thr1, Trp6, Trp7) was shared with phage clones c1, c10, c11 and c16. Although specific consensus sequences in all positions could not be identified from pattern enrichment, Thr in X_1_ and Trp in X_6_ were represented at particularly high frequencies (Fig. 5d). The most enriched patterns in CoV-2-c1 and c2 were found in the same position with similar residues (Fig. 5c and f). Interestingly, a reported peptide binder (P100) developed by a microarray platform^42^ contains the similar pattern ([*i*, *i* + 5, *i* + 6] = [T, W, M]) with our peptide hot spots (CoV-2-p1 [T, W, W] and p2 [T, W, F]). The P100 peptide is predicted from computer modelling to form a helical structure and attach to the ACE2-binding surface. Because patterns corresponding to clones c3 and c4 were ranked lower in the enrichment analysis, the phage clones c1, c2 and c5 were subjected to ELISA. As a control, a phage displaying ACE2(27–42) peptides, which weakly bind to the SARS-CoV-2 spike RBD, was also evaluated simultaneously. The results showed that CoV-2-c1, c2 and c5 phages bound significantly to the SARS-CoV-2 spike RBD (Fig. 5e).

These stapled peptides (CoV-2-p1, p2 and p5) were synthesised (Fig. S17–S19†). In CD measurements, CoV-2-p1, p2 and p5 exhibited 39%, 45% and 12% helical content, respectively, in 25% TFE, indicating that p1 and p2 prefer a helical conformation. The inhibitory activity of the synthesised peptides was examined by ELISA or pull-down competitive inhibition assay. From the ELISA competitive assay, we could not obtain reproducible results, presumably because of some technical issues (data not shown). In the pull-down assay, the biotinylated His-tag recombinant SARS-CoV-2 spike RBD was immobilised on resin, after which the mixture of His-tag recombinant ACE2 and stapled peptides was added. The band intensity of bound ACE2 decreased in a manner dependent on the peptide concentration (Fig. 5g and S20†). In addition, CoV-2-p1(3A) peptide, in which the three key residues of CoV-2-p1 were substituted with Ala did not inhibit the interaction (Fig. S21†). Thus, peptides containing the top position–amino acid pattern have the potential to inhibit the interaction between SARS-CoV-2 spike RBD and ACE2.

## Conclusions

Various peptide inhibitors developed by phage display have been used in clinical applications. However, this method is imperfect because the selection is strongly dependent on the conditions and inevitably suffers from compositional bias. There is thus a need to develop strategies to reduce the influence of bias and to reliably select clones that truly bind to the target.

In this study, we developed ‘pattern enrichment analysis’ in which large sequence datasets were obtained by NGS and compared them in terms of three-residue patterns. First, we validated the method by screening against HDM2, whose peptide ligands have been well defined. Three rounds of screening were conducted using a phage library tethering randomised stapled peptides. By counting repeating sequences, *i.e.*, the conventional method, clones that bind to HDM2 were rarely obtained. The clones with the highest counts were highly infectious, suggesting that the products were biased during the amplification stage. In contrast, pattern enrichment analysis revealed sequences similar to the known HDM2 ligand. Furthermore, key residues (Phe, Tyr, Trp and Leu) for the interaction were frequently detected at the appropriate spacing. These results indicate the usefulness of this approach for identifying the bound peptide sequences and also for determining hotspots. Next, three rounds of screening were conducted against SARS-CoV-2 spike RBDs, resulting in a significant decrease in diversity due to less nonspecific phage adsorption. The top patterns in the pattern enrichment analysis were parts of the top 1, top 2 and top 5 sequences. From the phage ELISA, these clones significantly attached to the SARS-CoV-2 spike RBD. Moreover, the synthesised peptides exhibited the inhibition of ACE2 binding to immobilised SARS-CoV-2 spike RBD. In both cases, the identified peptides exhibited a high propensity for forming helices. The key residues obtained from the pattern enrichment analysis could be mapped to the space of the helical structure, suggesting the advantage of using the structured peptide library in pattern enrichment analysis.

This analytical method is not limited to phage display, but is expected to be widely applicable to other genetically encoded libraries. We envision that, by appropriately setting the number of windows for pattern enrichment analysis, considering the diversity of the library and the number of NGS reads, the effects of bias can be greatly reduced with high coverage. Given the existence of hotspots in various protein–protein interactions, we expect that this method can be applied to a broad range of protein targets.

## Data availability

Experimental methods and detail results are provided in the ESI.†

## Author contributions

T. M., S. K. and K. S. convinced the project. T. M. constructed the phage library. K. N., S. K. and K. S. performed chemical modification of phage library, phage selection and ELISA assay. T. M., S. K., K. S. and K. N. made python manuscripts and analysed the FASTQ files. T. M. and G. M. synthesised peptides and tested the inhibition assay. T. M. and H. M. wrote the manuscript. All author made critical comments and contributed to the composition of manuscript.

## Conflicts of interest

There are no conflicts to declare.

## Supplementary Material

SC-013-D2SC04058A-s001
