# Supplementary material for: Pattern enrichment analysis for phage selection of stapled peptide ligands
Source: Chem Sci. 2022 Oct 12;13(43):12634–42. doi: 10.1039/d2sc04058a (PMC9645375; doi:10.1039/d2sc04058a)
Supplement: SC-013-D2SC04058A-s001 [file SC-013-D2SC04058A-s001.pdf]

Electronic Supplementary Material (ESI) for Chemical Science.
This journal is © The Royal Society of Chemistry 2022

Supporting information

Pattern enrichment analysis for phage selection of stapled peptide ligands

Takayuki Miki*, Keigo Namii, Kenta Seko, Shota Kakehi, Goshi Moro & Hisakazu Mihara

School of Life Science and Technology, Tokyo Institute of Technology, 4259 Nagatsuta-cho,

Midori-ku, Yokohama, Kanagawa 226-8501, Japan

Contents

Materials and Methods S3

Fig. S1 DNA sequence of the peptide fused gene-3 protein in the phage library S8

Fig. S2 NGS analysis and the processing of FASTQ data. S8

Fig. S3 DNA frequency at each position. S9

Fig. S4 Diversity plots of Amp-R1, R3, and R5 ..... S9

Fig. S5 Amino acid abundance at each position S10

Fig. S6 Chemical modification of peptides tethering on phages with the stapling linker 1. ..... S11

Fig. S7 The recovery ratio of phage during phage selection against HDM2 ..... S12

Fig. S8 Whole sequence comparison of HDM2-R3 with Amp-R3 ..... S13

Fig. S9 The result of the XSTREME motif analysis of HDM2-R3. ..... S14

Fig. S10 The distribution of $\log_{10}$(counts) value of each position-amino acid pattern in Amp-R3. ..... S14

Fig. S11 Comparison of the reported Ala-scanning results and the amino acid abundance calculated from our pattern enrichment study. S15

Fig. S12 Difference in stapling position between stapled p53 peptide and HDM2-p13/opt. S15

Fig. S13 Synthesised HDM2-p13 peptide S16

Fig. S14 Synthesised FAM-ATSP-3848 peptide S16

Fig. S15 Synthesised HDM2-opt peptide S17

Fig. S16 Screening of phage library against SARS-CoV-2 Spike RBD S17

Fig. S17 Synthesised CoV-2-p1 peptide S18

Fig. S18 Synthesised CoV-2-p2 peptide. S18

Fig. S19 Synthesised CoV-2-p5 peptide. S19

Fig. S20 Pull-down competition assay results of CoV-2-p1, p2, and p5. .... S19

Fig. S21 Pull-down competition assay results of CoV-2-p1(3A). S20

Table S1 Summary of NGS results and the processing (Amp-R1, R2, and R3) S21

Table S2 Summary of NGS results and the processing (HDM2-R1, R2, and R3). S21

Table S3 Summary of pattern enrichment analysis S21

Table S4 Summary of NGS results and the processing (SARS-CoV-2 Spike RBD-R1, R2, and R3). S21

Table S5 A list of synthesised peptides against HDM2. .... S22

Table S6 A list of synthesised peptides against SARS-CoV-2 Spike RBD S22

Reference S22

Materials and Methods

Preparation of XC6CX phage library.

We designed the fd phage library tethering XC6CX (XCXXXXXXCX; X, randomized residue; C, Cys) at N-terminus of the minor coating protein pIII. Here, we used a *fdg3p021* phage vector, which was mutated to contain disulfide-free minor coat protein pIII. The phage vector was kindly gifted from Prof. Henis. According to the manuscript from Prof. Heinis and co-workers[1], we prepared the library by the following procedure; (1) preparation of DNAs coding randomized peptide, (2) ligation and transformation and (3) phage preparation.

Firstly, a gene coding domains D1 and D2 of cysteine free gene-3 protein was amplified by PCR. Then, the product was purified by agarose electrophoresis and DNA extraction using NucleoSpin® Gel and PCR Clean-up kit (MACHEREY-NAGEL GmbH & Co. KG).

Forward primer; GGCGGATCCGGCGCTGAAACTGTTGAAAGTAG

Reverse primer; GAAGCCATGGCCCCCGAGGCCCCGGACGGAGCATTGACAGG

For the preparation of DNA fragments cording randomized peptide fused D1-2 domains of gene-3 protein, we performed PCR using the 1st PCR product as the template and purified.

Forward primer; TATGCGGCCCAGCCGGCCATGGCA<u>NNKTGTNNKNNKNNKNNKNNKNNKTGC NNK</u>GGTGGCTCTGAAAACCTGTACTTCCAATCCGGCGGATCCGGCGCTG

Reverse primer; GAAGCCATGGCCCCCGAGGCCCCGGACGGAGCATTGACAGG

The *fdg3p021* phage vector was digested by EcoRI and SfiI, and purified by agarose electrophoresis and DNA extraction from the gel. In the same manner, the 2nd PCR product was digested by SfiI and purified. The DNA fragment was inserted into the linearized vectors by T4 DNA ligase (New England Biolabs). To desalt the mixture, the buffer was exchanged to ultra-pure water by repeats of ultrafiltration. The ligation product was introduced to competent TG-1 cells (Lucigen) by electroporation. The transformed cells were incubated on 2-YT agar containing chloramphenicol. The cells were collected and stocked in 15% glycerol. Then, we finally obtained a fd phage library tethering XC6CX (XCXXXXXXCX; X, randomized residue; C, Cys) at N-terminus of the minor coating protein pIII with 1.4×10^9 (t.u. transducing unit) diversity.

Modification of XC6CX phage library

Compound **1** was synthesised in the same manner as our previous report[2]. The phage library (10-20 nM) was reduced in 50 mM HEPES buffer (pH 8.0) with 1 mM TCEP (*Tris*(2-carboxyethyl)phosphine Hydrochloride) at 42 ºC for 1 hour. To remove TCEP, the buffer was exchanged by the repeat of ultrafiltration (Amicon Ultra, NMWL:10K, Merck) into 50 mM HEPES buffer (pH 8.0). The reduced phages were modified with 50 μM of compound **1** for 3 hours at 42 ºC. To remove the excess reagents, we precipitated the phages by addition of 20%v/v of 20%PEG6,000/2.5M NaCl solution and incubation at 4 ºC for 1 hour. The precipitate was obtained by centrifugation (9,000 g, 30 min at 4 ºC) and dissolved in 50 mM HEPES (pH 8.0) buffer. We repeated the purification step. The final concentration of phage was determined by UV absorbance (269 nm).

NGS analysis

We purified the phage vectors from infected TG-1 cells by using FastGene PlasmidMini (NIPPON Genetics). The

preparation and sequencing for NGS analysis were conducted by FASMAC (Japan). The concentration was determined by Qubit. The dsDNA fragments were prepared by PCR (polymerase chain reaction). At the first PCR, the 197 bp including the region coding peptide was amplified by ExTaq DNA polymerase from the phage vector pool using primers (Forward; ACACTCTTTCCCTACACGACGCTCTTCCGATCTTTGGAGATTTTCATCATGA, Reverse; GTGACTGGAGTTCAGACGTGTGCTCTTCCGATCTCCTTCTATATGGGATTTTG). The PCR products were purified by AMPure XP (Beckman Coulter) beads. The 2 μL of products were used for the second PCR, the products were purified by AMPure XP beads. The quality was checked by Bio 2100 (High Sensitivity DNA). The samples were subjected to Illunina MiSeq (250 bp, pair-end reads). We obtained FASTQ files including more than 100,000 reads for each sample. For Amp-R1 and Amp-R3, we conducted the sequencing triplicates. The triplicates were combined into single file after processing.

Processing of FASTQ files obtained from NGS

The data processing was performed based on Heinis' and Derda's reports[3,4]. For each sample, the two raw data (FASTQ format) was obtained from Illumina MiSeq sequencing. Using a Python (3.7.7) with Biopython (1.78) and Pandas (1.2.4) packages and Jupyter Notebook (6.4.0), we processed the raw files to write a FASTA file containing peptide sequences of interest regions.

a. Trimming the DNA region of interest: The region 30 bp corresponding to the randomized peptide (XCXXXXXXCX) was inserted between 'GGCCCAGCCGGCCATGGCA' and 'GGTGGCTCTGAAAACCTG' (Fig. S1). For each read, the 30 bp region was searched with the two sequences; if the region of a read is not match with 30 bp, the read was excluded.
b. Extraction of reads match with design: We extracted DNA sequences matching with the designed sequence 'NNKTGTNNKNNKNNKNNKNNKNNKTGCNNK' (N; A, T, G or C, K; T or G).
c. Quality filter: To collect reliable sequences, the sequences containing three or more residues with a Q-value of less than 30 were excluded.
d. Collection of pair-matched sequences: For the reverse reads in the pair-end sequencing, reverse-complement was conducted, followed by the process a–c. The two processed reads were compared, and the completely-matched sequences were collected.
e. Translation of the 30 bp DNA to a peptide sequence.
   Because of Amber suppressor strain (supE) of TG1 bacterial cells, UAG was translated into glutamine.

From these processes, 74–84% reads in raw data was remained. As for Amp-R1 and Amp-R3, we triplicated the sequencing and combined three processed FASTA files into a single FASTA file.

Selection of stapled peptide ligands

Recombinant HDM2 protein was expressed and purified by His-tag affinity columns (TALON®, Clontech) resin as described in our previous report[2]. The 100 μL of purified HDM2 (100 μg/ml) per well was placed on 96 well plate (Costar® Assay Plate 3923. flat bottom, high binding) and incubated for overnight at 4 °C to immobilize the protein. After rinsing with HBS-T buffer (10 mM HEPES, 150 mM NaCl, 0.1% v/v tween-20, pH 7.5) three times, the wells were incubated with 300 μL of blocking buffer (HBS-T containing 1%w/v BSA) for 1 hour at room temperature.

Then, the wells were rinsed with HBS-T for three times. At the same time, the purified stapled phage library was mixed with 4×blocking buffer (10 mM HEPES, 150 mM NaCl, 4% w/v BSA, 0.2% Tween-20) and incubated for 30 min at room temperature to afford the blocked phage library. To the two immobilized wells, the 600 μL (300 μL/well) of blocked stapled phage solutions (8.2 nM, 4×10^{10} t.u.) were added and incubated for 1 hour at room temperature. After washing with (300 μL/well) HBS-T for ten times, the bound phages were eluted with 400 μL (200 μL/well) of Gly-HCl buffer (50 mM, pH 2.2) followed by 400 μL (200 μL/well) HBS-T. The eluents were neutralized by 100 μL of 1M Tris-HCl (pH 8.0). The eluted phages were added to 25 mL of TG-1 *E. coli* culture (OD_{600} = 0.4) and infected for 90 min at 37 ºC. We harvested the cells by centrifugation (4,000 rpm, 5 min, 4 ºC) and removed the supernatant. The pellets were suspended with 1 mL of 2×YT medium, seeded on a 2×YT agar (15 cm diameter plate, containing 20 μg/mL chloramphenicol) and incubated overnight at 37 ºC. The colonies were suspended in 4 mL of 2×YT medium and mixed with 1 mL 50% glycerol. The suspension was divided in ten tubes and frozen in liquid nitrogen and stocked in -80 ºC. For second and third rounds, stapled phage pool amplified from 1st round (6.0 nM, 600 μL, 6×10^{9} t.u.) and stapled phage pool from 2nd round pool (2.7 nM, 600 μL, 4×10^{9} t.u.) were used, respectively.

For selection against SARS-CoV-2 Spike RBD, the 100 μL of recombinant SARS-CoV-2 Spike RBD (318-541) (48801S, Cell Signaling Technology) solution (100 μg/mL) were immobilized on a 96-well plate for overnight at 4 ºC. After rinsing with HBS-T three times, the well was blocked by one-hour incubation of HBS with 1%w/v BSA and rinsed with 300 μL of HBS-T three times. The stapled phage library (10 nM) was incubated in HBS with 1%w/v BSA for 30 minutes, and 200 μL of blocked phages (10 nM, 2 pmol, 2×10^{10} t.u.) were added to the plates. After one hour incubation, the plates were washed with HBS-T for five times. The bound phages were eluted with 200 μL of Gly-HCl buffer (200 mM, pH 2.2) followed by 200 μL of HBS-T and neutralized by 50 μL of 1M Tris-HCl (pH 8.0). The phages infected to TG-1 cells and, we made glycerol stocks as described above. At the second round, the 10 μg of protein (100 μg/ml, 100 μL) was immobilized and 0.9 pmol of blocked phage library (10 nM, 90 μL, 8×10^{9} t.u.) were used, and we washed HBT-T for ten times to remove unbound phages. At the third round, the 2 μg of protein (20 μg/ml, 100 μL) was immobilized and 1.0 pmol of blocked phage library (10 nM, 100 μL, 6×10^{9} t.u.) were used, and we washed HBT-T for ten times to remove unbound phages. The phage vector pools were subjected to NGS analysis.

ELISA of phage clones binding to HDM2 or SARS-CoV-2 Spike RBD

On a Corning 96-well black flat bottom polystyrene high bind plate, recombinant HDM2 (300 μg/mL, 100 μL per well, 3 μg per well) in 10 mM HEPES buffer (pH 8.0, containing 5 mM EDTA) was incubated for overnight at 4 ºC and rinsed with HBS buffer (10 mM HEPES, 150 mM NaCl, pH 7.5) for three times. For blocking, the well was incubated with 1w/v% BSA HBS buffer (200 μL) for one hour at room temperature, followed by washing with HBS-T three times. Chemically modified phage clones (1 nM, 100 μL) were added to the well and incubated for one hour at room temperature. The unbound phages were removed by washing with HBS-T for five times. The wells were incubated with an anti-M13 antibody HRP-conjugates (1:2000 dilution, mouse monoclonal, SinoBiological, 11973-MM05T-H) in HBS-T for one hour at room temperature. After rinsed with HBS-T (three times), 100 μL of QuantaBlu working solution was added. After 45 min incubation, stop solution was added and the fluorescence intensities were measured by ARVO MX plate reader with Wallac1420 Workstation (Excitation, 355/40-nm F355 filter; Emission,

460/40-nm F460 filter).

For the ELISA against CoV-2 Spike RBD, a 100 μL of recombinant SARS-CoV-2 Spike RBD (318-541) (48801S, Cell Signaling Technology) solution (3 μg/mL, 100 μL per well, 0.3 μg per well) was immobilized on a plate. For binding step, modified phage clones were incubated at 2 nM concentration. The other steps were exactly same as ELISA against HDM2.

Three-window pattern enrichment analysis

The raw FASTQ files resulting from pair-end sequencing using Illumina MiSeq were processed to obtain FASTA files containing peptide sequences with high quality. For each dataset (single FASTA file), we calculated the abundances of each position-amino acid pattern by the following steps. Among eight random residues (X1, X3-X8, X10), select three residues (e.g. [X1, X3, X4]). For each of 8,000 (20^3) three-window amino acid patterns in that position pattern, we counted the number of sequences containing the sequence pattern. For sequence patterns that resulted in a zero count, a value of 0.8 was assigned. The abundance was calculated by dividing the counts of each pattern by the total counts. This procedure is performed for all 56-position patterns (${}_8C_3$) to obtain abundance values for all pattern-sequence patterns (448,000 patterns). The enrichment of each position-amino acid pattern was determined by the ratio of the abundance in the pools screened (HDM2-R3 or SARS-CoV-2-spike RBD-R3) to that in Amp-R3 (Table. S3).

Peptide synthesis

The peptides were synthesized on TentaGel S-RAM resins (HiPep Laboratories) by a standard Fmoc-solid phase peptide synthesis using Initiator+Alstra (Biotage). For deprotection, the resins were incubated in 20% piperidine (PPD) NMP (*N*-methyl-2-pyridone) at room temperature. For elongation, 4 eq. of Fmoc amino acid, 4 eq of HBTU, 4 eq of HOBt and 8 eq of DIPEA were used. Except for Cys and His elongation, the reactions were performed at 75 ºC for 5 min. For FAM-ATPS-3484 synthesis, diacetyl 5-carboxyfluorescein NHS ester was attached to the N-terminus, followed by deprotection with 20% PPD NMP. The peptides were deprotected and eluted by TFA(Trifluoroacetic acid)/H_2O/Triisopropylsilane (95:2.5:2.5) mixture. The eluate was precipitated in cold diethyl ester and dried up. The peptides were dissolved in 20 mM HEPES/acetonitrile (pH 8.0) at 1.0 mM and were reacted with compound **1** (1.5 eq) for two hours at 37 ºC. The stapled peptides were purified by RP-HPLC (Chromaster®, HITACHI) equipped with COSMOSIL 5C18-AR-II packed column (10 × 250 mm). We used 0.1% TFA water and 0.08% TFA acetonitrile mixture as mobile phase (Flow rate, 3.0 mL/min; linear gradient). Purified peptides were identified by MALDI-TOF MS (UltrafleXtreme, Bruker Daltonics) and lyophilized. Purity check was performed by RP-HPLC. The concentration of peptide stocks in DMSO or HBS buffer were determined by absorbance at 280 nm.

CD measurements

Peptide stocks in HBS buffer (10mM HEPES, 150 mM NaCl, pH 7.5) were diluted to 30 μM final concentration with HBS buffer or TFE (2,2,2-trifluoroethanol). The peptide solutions were placed in a quartz cell (1.0 mm wavelength). We obtained CD spectra by using a J-1100 (JASCO) spectropolarimeter with spectra manager (2.8.0.4). The helical contents were calculated by BeStSel web server[5].

Competition binding assay

A fluorescence polarization assay was performed to assess inhibition capability of HDM2-p13 and HDM2-opt peptides. The peptides (50 nM–50 μM) was mixed with 100 nM of HDM2 and 10 nM of FAM-ATPS-3848 in HBS buffer (10 mM HEPES, 150 mM NaCl, pH 7.5). The fluorescence polarization was measured by ARVO MX plate reader with Wallac1420 Workstation (Excitation, 355/40-nm F355 filter; Emission, 460/40-nm F460 filter). IC_{50} values of peptide inhibitors were calculated by nonlinear regression analysis using Prism (GraphPad Software).

Pull-down competition binding assay

Recombinant SARS-CoV-2 Spike RBD His Biotin Protein (R&D Systems, BT10500) at 4 μg/mL concentration in HBS buffer was incubated with 3 mg/mL of DynaBeads M-280 (ThermoFisher) for 2 hours at 4 ºC. After rinsed with HBS buffer, the immobilised beads were divided equally into the number of samples. To the beads (0.15 mg) was added 50 μL mixture of His tag ACE2 protein (Hakarel, HAK-ACE2_UL-1) (30 μg/mL) and peptides (0–100 μM). After one hour incubation at room temperature, the beads were rinsed three times with HBS-T (200 μL/well). Bound proteins were eluted in 50 μL of 3xLammli buffer with 2 mM biotin by boiling for 5 min. The samples were subjected to SDS-PAGE electrophoresis and transferred onto a PVDF membrane. After blocking with 2% skimmed milk TBS-T for 30 min at room temperature, the membrane was incubated in the HRP-conjugated His-tag monoclonal antibody (proteintech, HRP-7705, 1/1,000 dilution) TBS-T solution for overnight at 4 ºC, rinsed with TBS-T and soaked in Amersham ECL prime reagents (Cytiva). The chemiluminescent bands were detected using LuminoGraphI (ATTO) with ImageSaver6 software. From the band intensities, IC_{50} values of peptide inhibitors were calculated by nonlinear regression analysis using Prism (GraphPad Software).

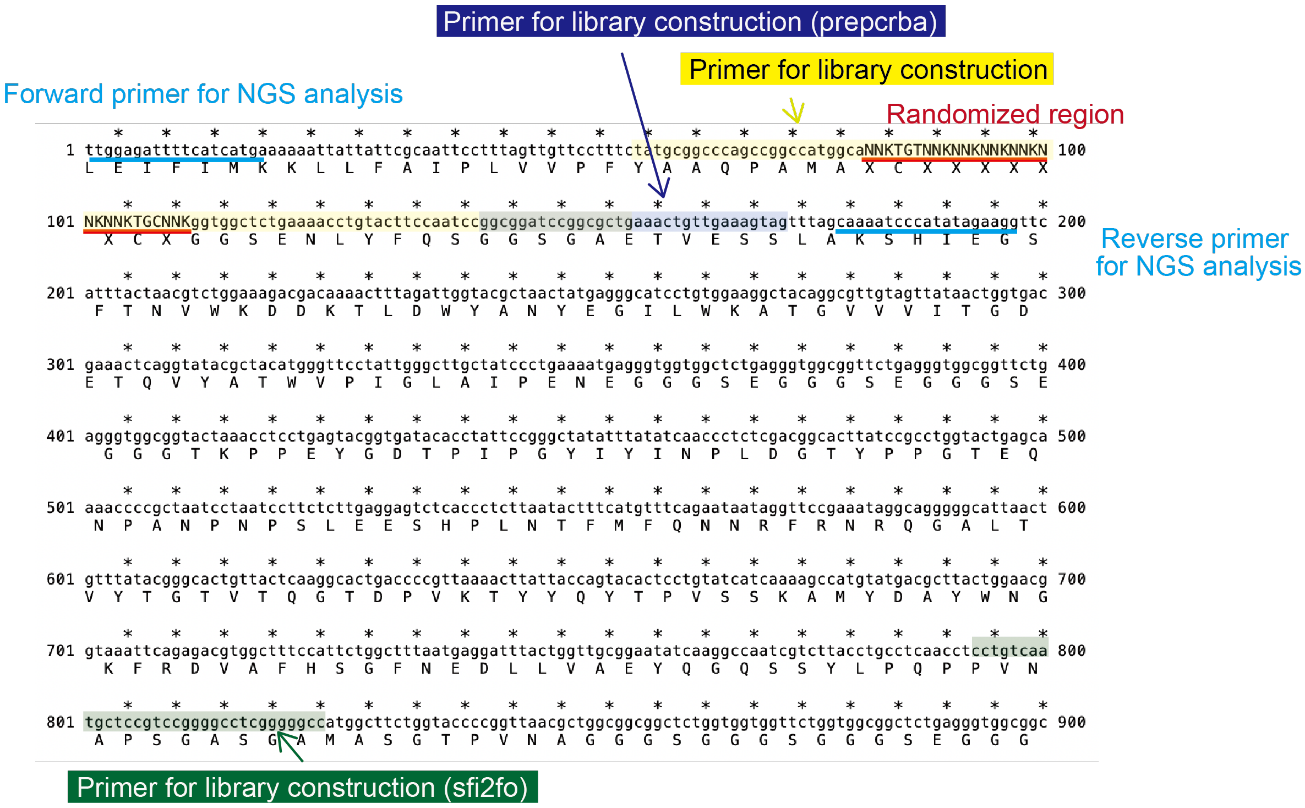

Figure S1. DNA sequence of the peptide fused gene-3 protein in the phage library. For the construction of the phage library, *prepcrba* and *sfi2fo* primers were used for the 1st PCR. To introduce randomised peptide, the *sfi2fo* primer and sequences highlighted in yellow were used for the 2nd PCR product, where the 1st PCR product was used as the template. Two primers underlined in blue were employed for the NGS analysis.

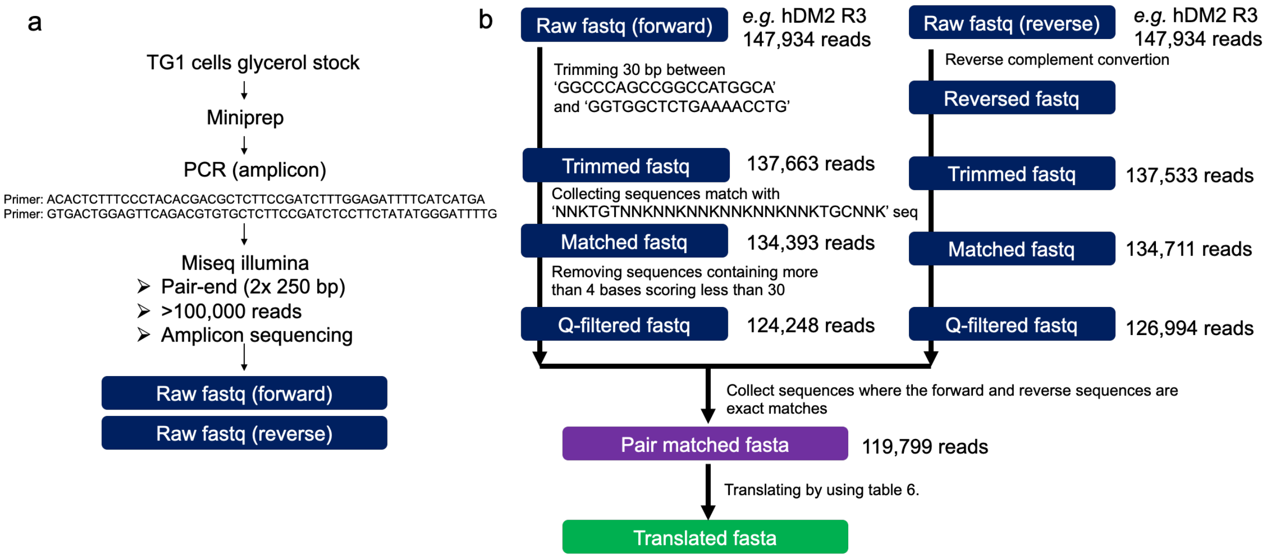

Figure S2. NGS analysis and the processing of FASTQ data. (a) The procedure of NGS analysis. From the infected TG-1 cells, phage vectors were extracted using FastGene Plasmid Mini Kit (NIPPON Genetics Co., Ltd.). The purified vectors were subjected to amplicon sequencing using custom primers. (b) Frow chart of the FASTQ data processing.

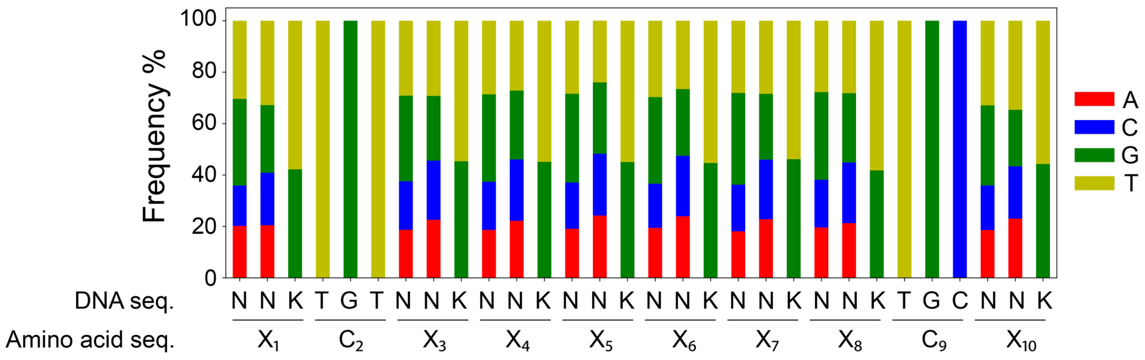

Figure S3. DNA frequency at each position. In Amp-R1, the nucleotides in 'NNK' codons were ideally inserted.

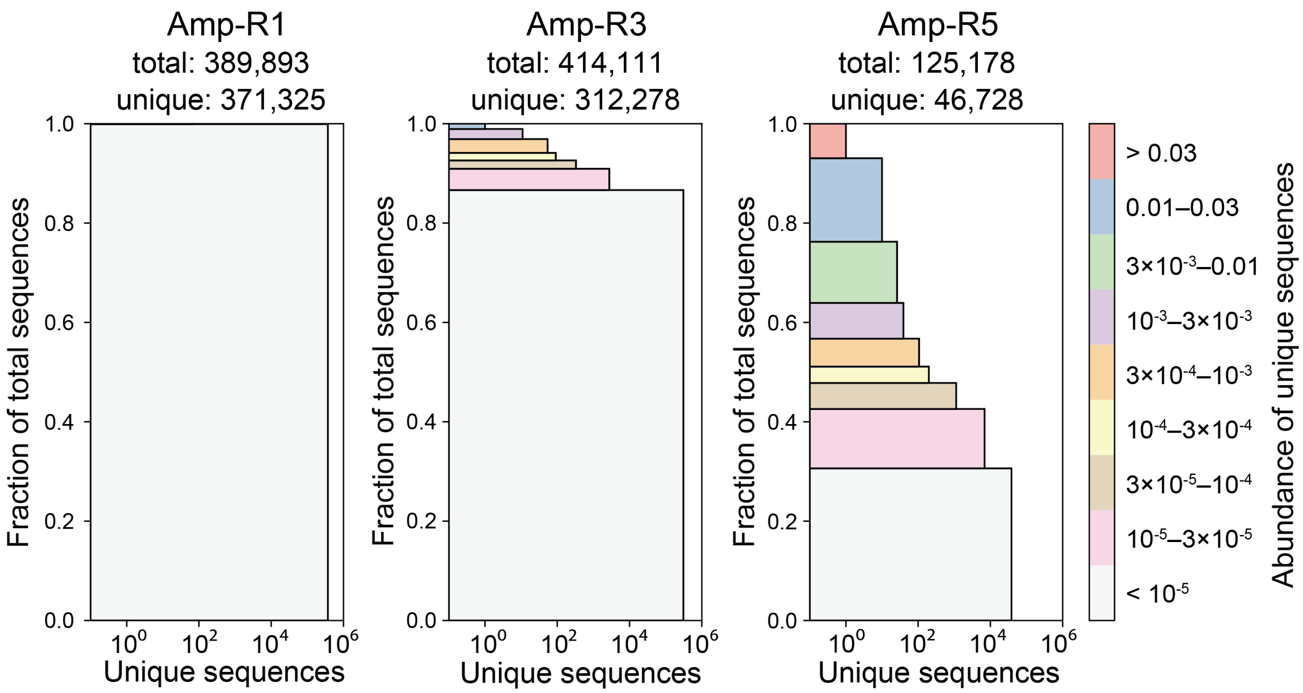

Figure S4. Diversity plots of Amp-R1, R3, and R5. As increasing of amplification cycles, the peptide diversity was dramatically decreased.

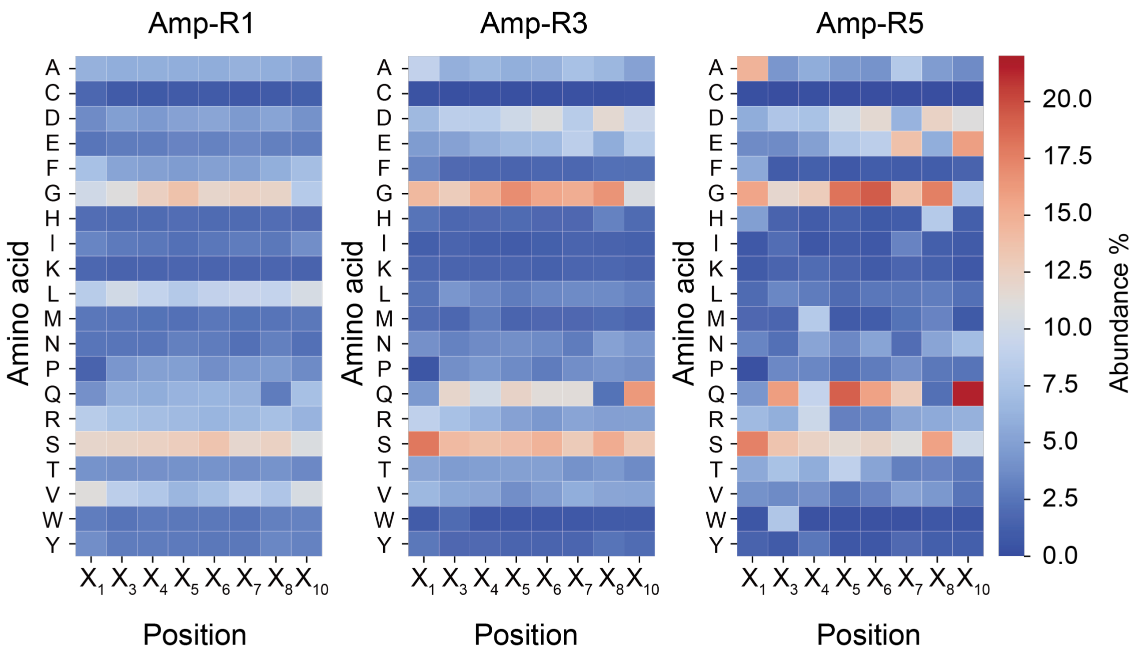

Figure S5. Amino acid abundance at each position. In Amp-R1, Leu and Ser were found in high frequency due to the codon redundancy. In Amp-R3 and R5, Gly and hydrophilic residues such as Ser, Gln, Asp, and Glu were enriched while hydrophobic residues (Trp, Tyr, Leu, Ile, Phe, and Val) and Cys were decreased.

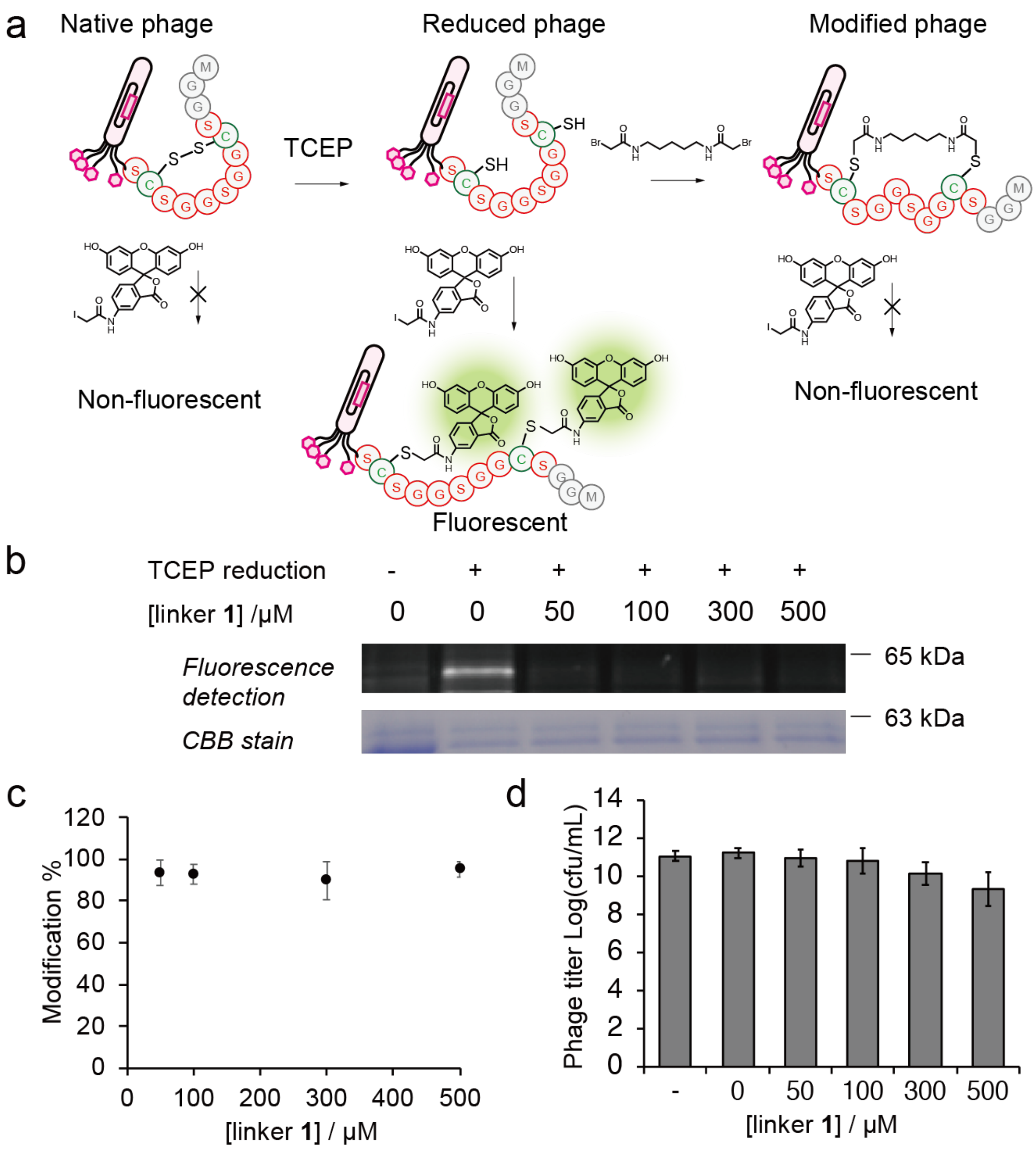

Figure S6. Chemical modification of peptides tethering on phages with the stapling linker 1. (a) Schematic illustration of quantification of modification yield. Firstly, phages were reduced by TCEP (1 mM) and purified by ultracentrifugation. The reduced phages were incubated with the linker **1** (0–500 μM), followed by mixing with 5-IAF (5-iodoacetamide-fluorescein) to visualize the unreacted Cys residues. (b) A fluorescence gel image of fluorescein-labeled gene-3 protein. The fluorescent band corresponding to gene-3 protein was diminished by adding **1**. (c) The modification yields. (d) Phage titer after modification. Phage function was less susceptible to chemical modification. The phage titer was gradually decreased at 300 μM of **1**. (n = 3, error bar means SD)

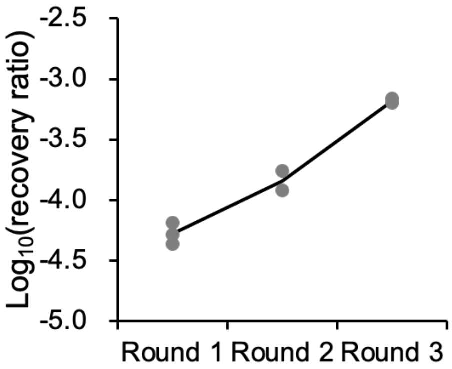

Figure S7. The recovery ratio of phage during phage selection against HDM2. As increasing the selection rounds, the recovery ratio was increased.

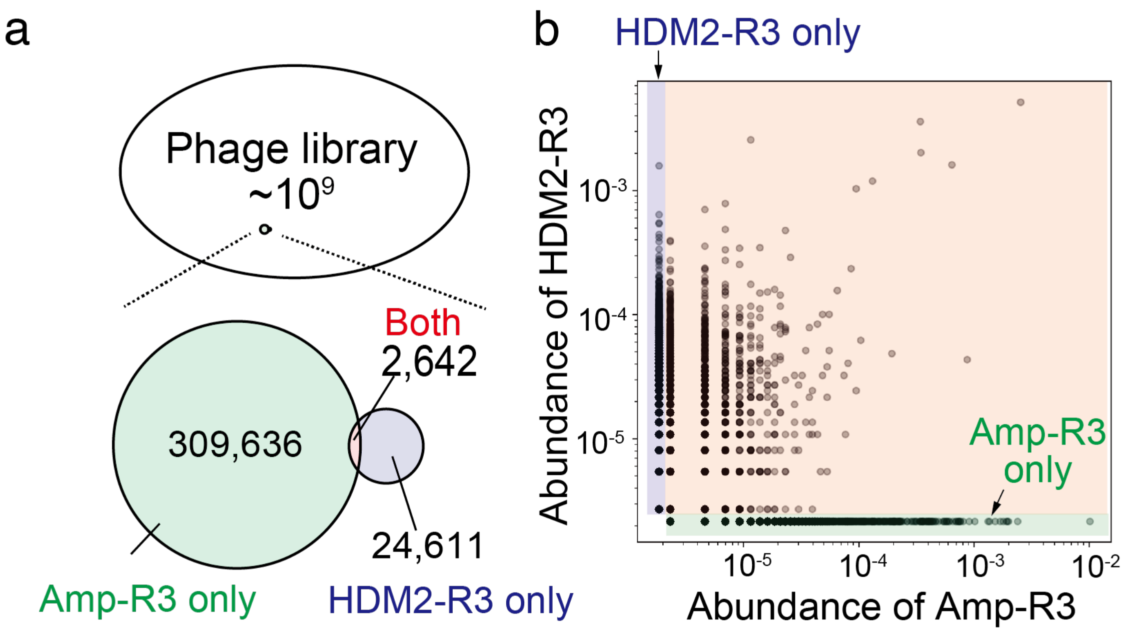

c

Phage	X1	C2	X3	X4	X5	X6	X7	X8	C9	X10	Ratio	HDM2-R3 Counts	Amp-R3 Counts
HDM2-p6	Q	C	F	A	Q	S	V	G	C	P	859	582	0.8
HDM2-p11	R	C	P	G	S	S	N	S	C	D	348	236	0.8
HDM2-p12	L	C	N	N	S	D	V	F	C	S	298	202	0.8
HDM2-p13	A	C	F	D	E	Y	W	Q	C	L	294	199	0.8
HDM2-p16	V	C	S	S	V	K	K	C	C	P	242	164	0.8
HDM2-p17	N	C	F	G	D	Y	W	D	C	I	229	155	0.8
HDM2-p3	R	C	S	V	S	N	Q	A	C	S	224	949	5
HDM2-p19	G	C	S	S	G	Q	K	G	C	S	211	143	0.8
HDM2-p24	N	C	E	Y	E	G	W	D	C	N	183	124	0.8
HDM2-p18	R	C	G	S	E	V	Q	G	C	P	172	146	1
			i				*i+4*			*i+7*			

Figure S8. Whole sequence comparison of HDM2-R3 with Amp-R3. (a) A Venn diagram of sequence coverage. NGS analysis reveals a little part of whole sequences. The majority of sequences in HDM2-R3 could not quantified. (b) Scattering plot of sequence abundances of Amp-R3 and HDM2-R3. (c) Top 10 sequences in the whole sequence enrichment analysis. The c13 contains FWL hydrophobic triad. The c17 contains F and W at *i* and *i+4* positions.

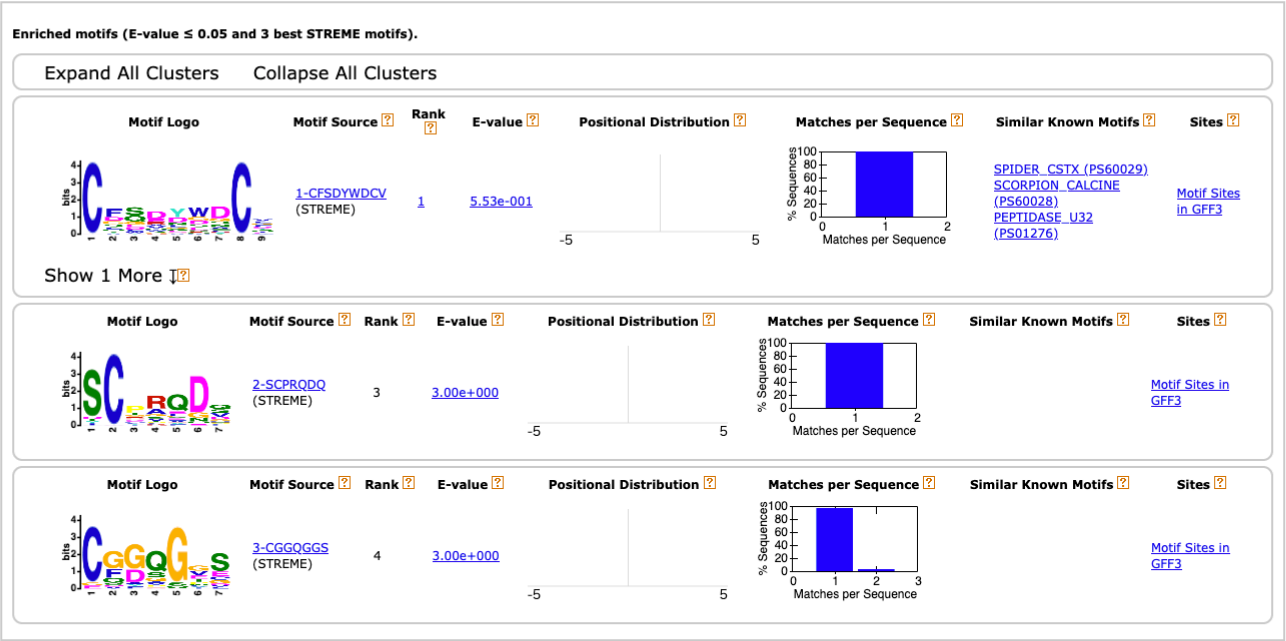

Figure S9. The result of the XSTREME motif analysis of HDM2-R3. The Amp-R3 dataset was used as a control sequence. The STREME search[6] was stopped after 3 motifs whose *E*-values exceeded 0.05 had been found.

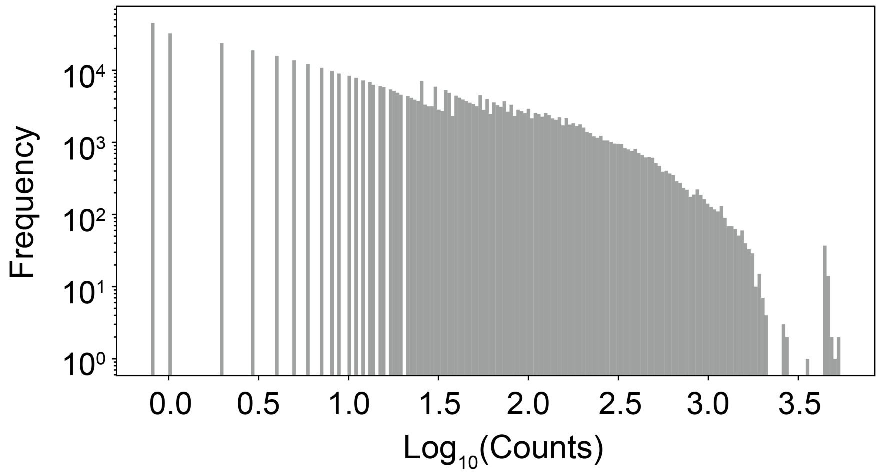

Figure S10. The distribution of $\log_{10}$(counts) value of each position-amino acid pattern in Amp-R3. For each position-amino acid pattern (448,000 patterns), the sequences matching the pattern were counted in Amp-R3 dataset.

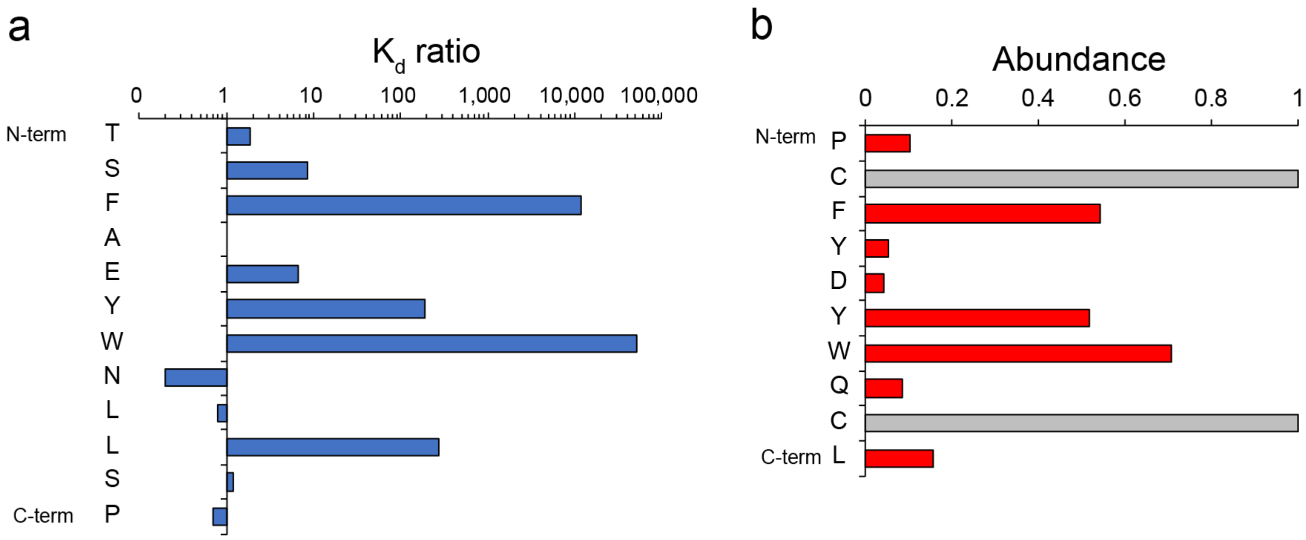

Figure S11. Comparison of the reported Ala-scanning results and the amino acid abundance calculated from our pattern enrichment study. (a) The reported K_d ratio values of Ala-substituted peptides to the original peptide[7] (PWI peptide; TSFAEYWNLLSP). (b) Amino acid abundancy of HDM2-opt at each position. Red bars correspond to randomised positions.

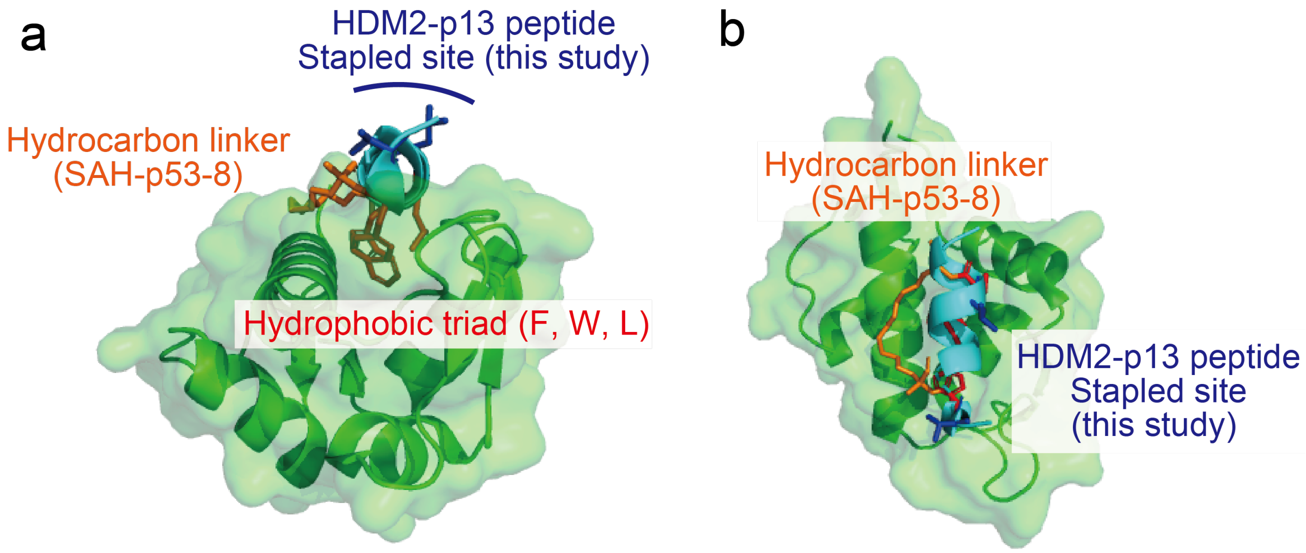

Fig. S12 Difference in stapling position between stapled p53 peptide and HDM2-p13/opt. The X-ray structures of MDM2 and SAH-p53-8 complex[8] (PDB: 3V3B) were shown in (a) side view and (b) top view. (Red; Hydrophobic triad. Orange; hydrocarbon stapling linker of SAH-p53-8, Blue; the residues corresponding to stapled sites of HDM2-p13 and -opt).

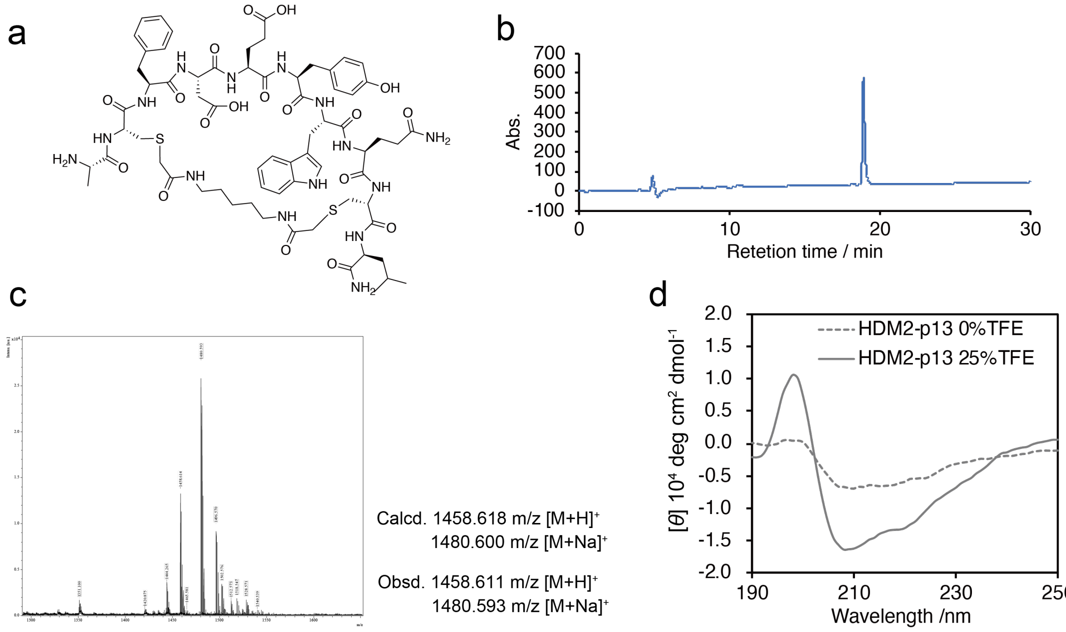

Fig. S13 Synthesised HDM2-p13 peptide. (a) Chemical structure of HDM2-p13. (b) Analytical HPLC for purity check. (c) MALDI-TOF MS results. (d) CD spectrum of HDM2-p13 (dashed line; in absence of TFE, straight line; in presence of 25% TFE)

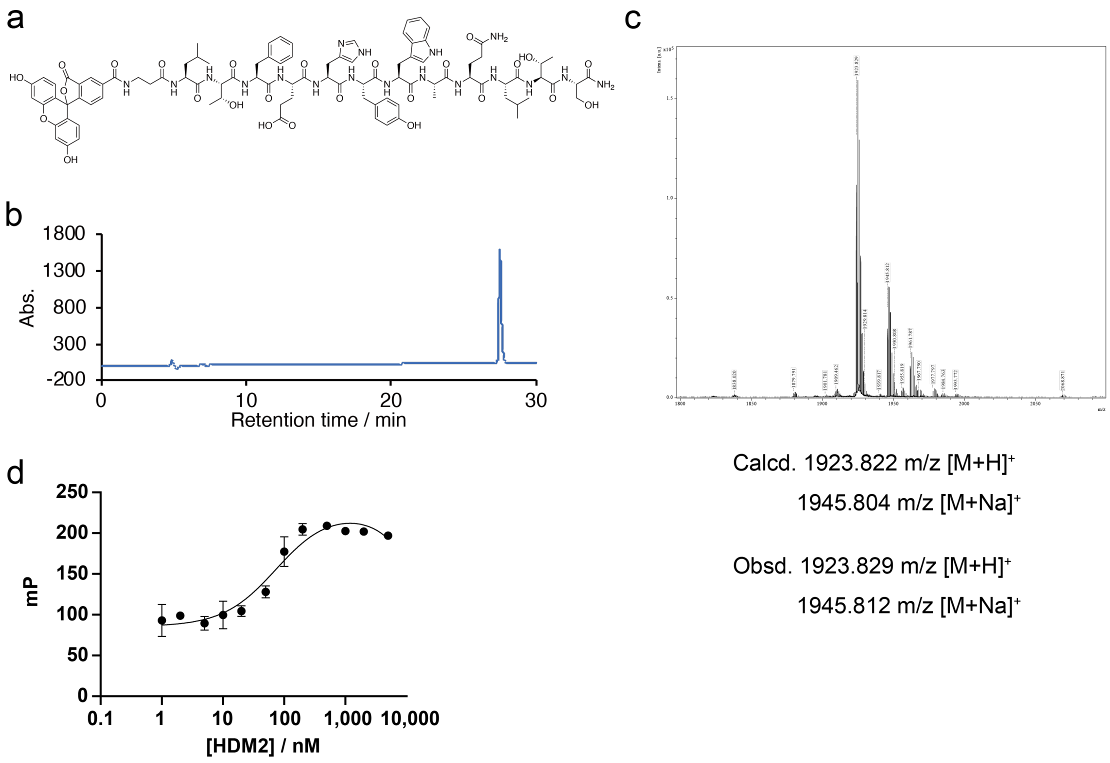

Fig. S14 Synthesised FAM-ATSP-3848 peptide. (a) Chemical structure of FAM-ATSP-3848. (b) Analytical HPLC for purity check. (c) MALDI-TOF MS results. (d) Fluorescence polarization assay[9] of FAM-ATSP-3848 binding to HDM2. The concentration of FAM-ATSP-3848 was fixed to 10 nM, the HDM2 concentration was varied from 1–3,000 nM (n = 3, error bars mean SD).

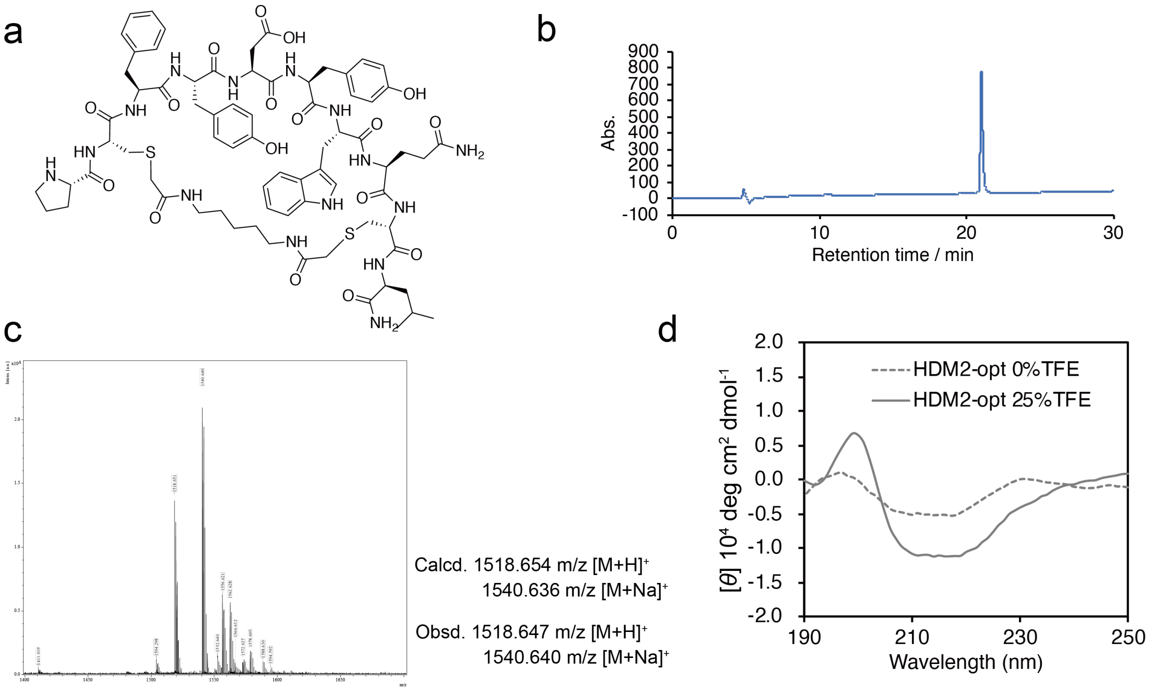

Fig. S15 Synthesised HDM2-opt peptide. (a) Chemical structure of HDM2-opt. (b) Analytical HPLC for purity check. (c) MALDI-TOF MS results. (d) CD spectrum of HDM2-opt (dashed line; in absence of TFE, straight line; in presence of 25% TFE)

a

Round	1st	2nd	3rd
Immubilized protein / μg	10	10	2
Phage library/ pmol	2	0.9	1
HBS-T Wash	5 times	10 times	10 times

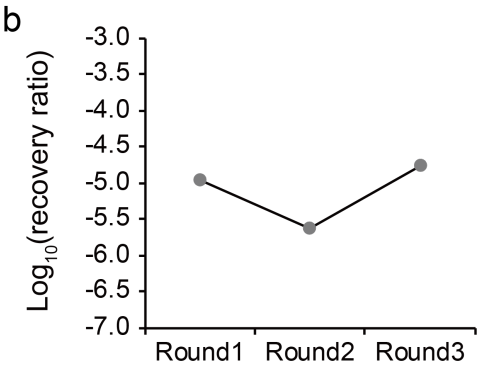

Fig. S16 Screening of the phage library against SARS-CoV-2 spike RBD. (a) Conditions of phage selection. (b) Recovery yields in each round.

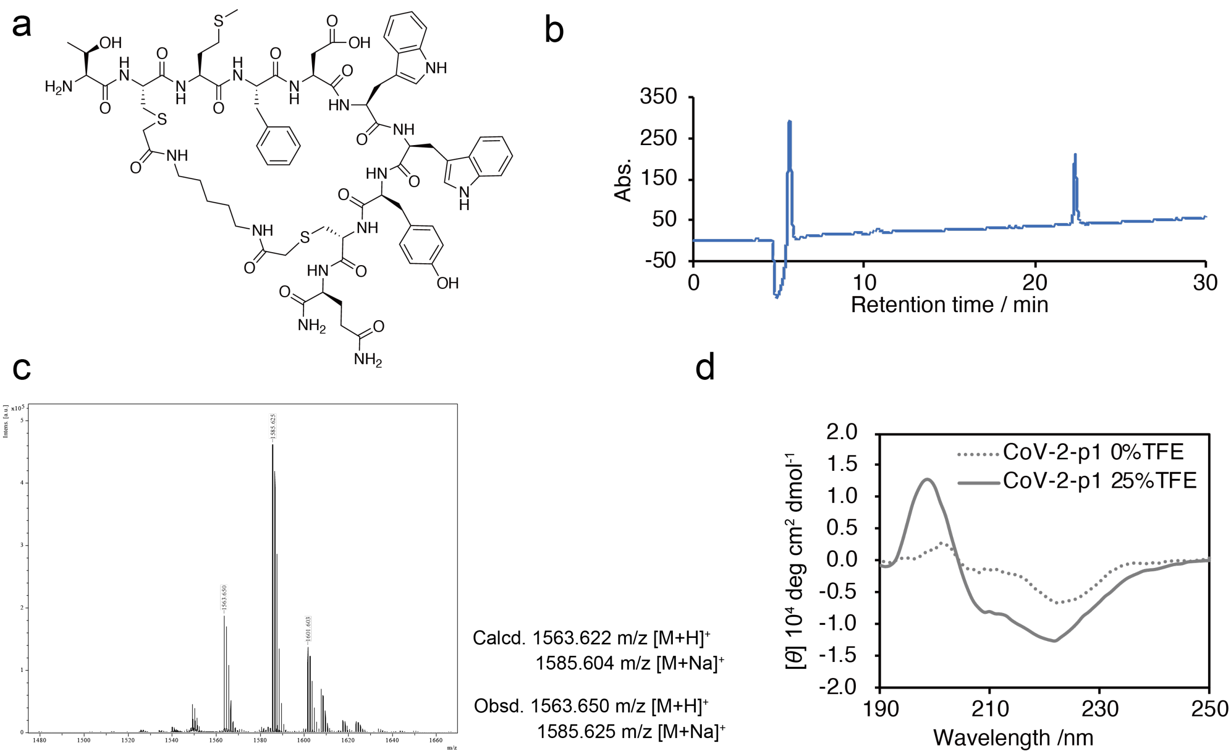

Fig. S17 Synthesised CoV-2-p1 peptide. (a) Chemical structure of CoV-2-p1. (b) Analytical HPLC for purity check. (c) MALDI-TOF MS results. (d) CD spectrum of CoV-2-p1 (dashed line; in absence of TFE, straight line; in presence of 25% TFE)

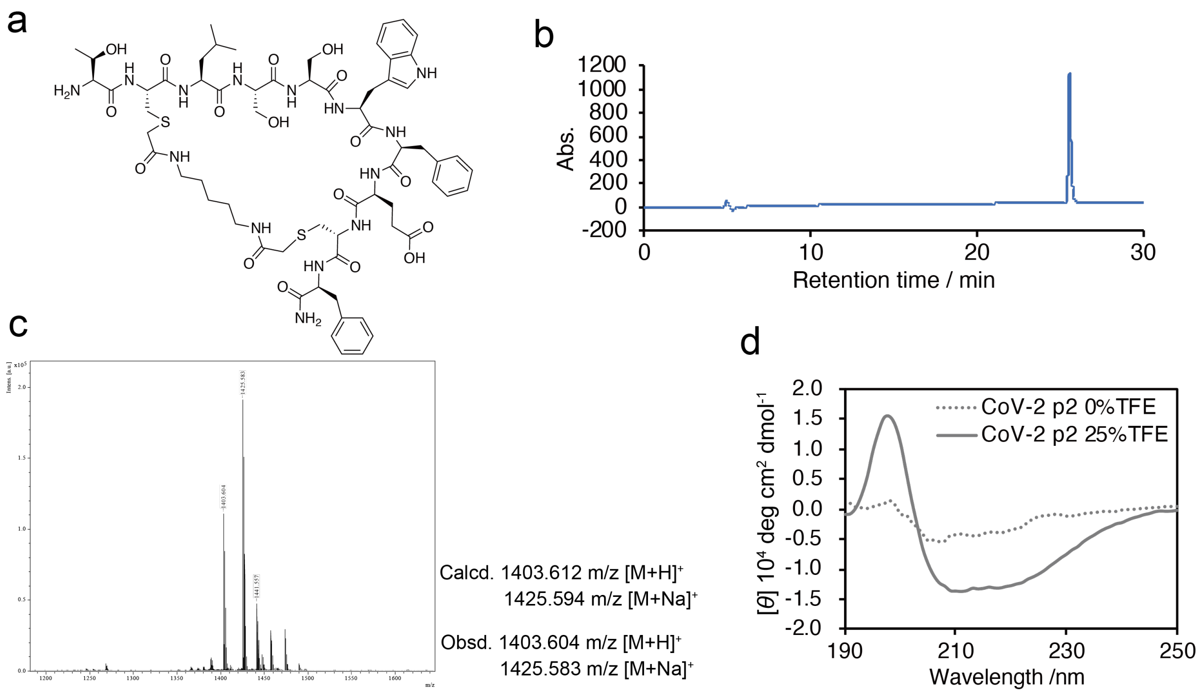

Fig. S18 Synthesised CoV-2-p2 peptide. (a) Chemical structure of CoV-2-p2. (b) Analytical HPLC for purity check. (c) MALDI-TOF MS results. (d) CD spectrum of CoV-2-p2 (dashed line; in absence of TFE, straight line; in presence of 25% TFE)

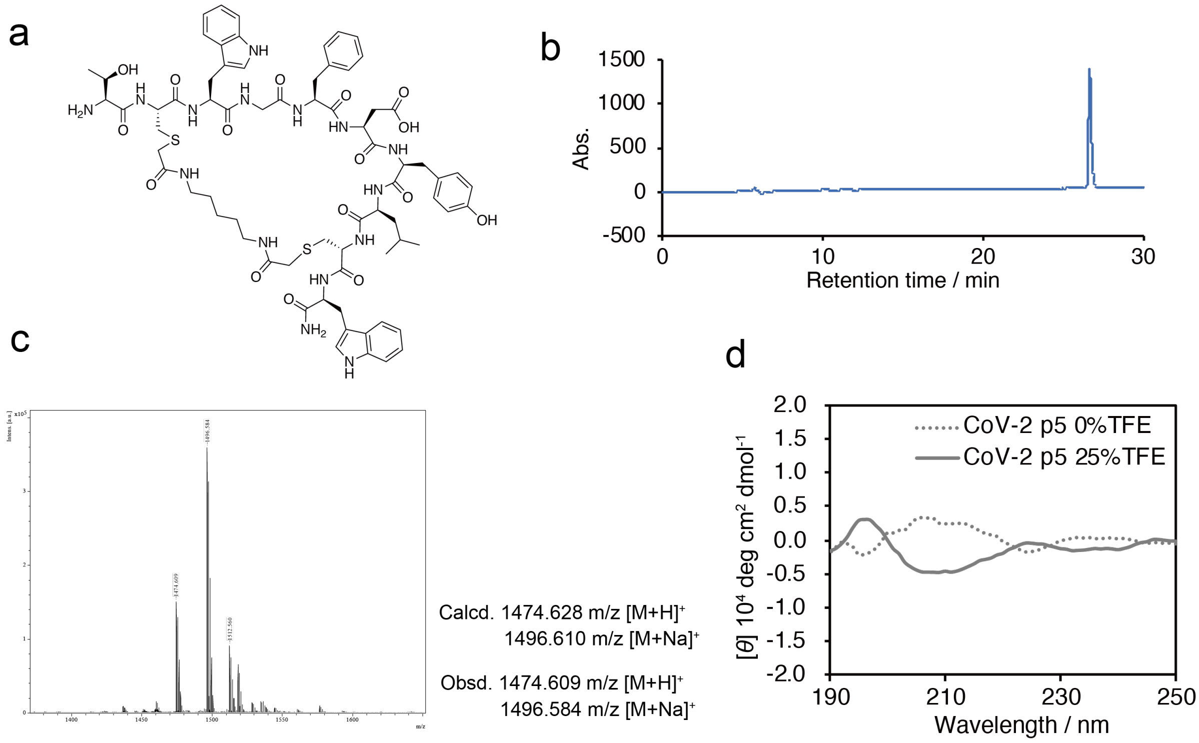

Fig. S19 Synthesised CoV-2-p5 peptide. (a) Chemical structure of CoV-2-p5. (b) Analytical HPLC for purity check. (c) MALDI-TOF MS results. (d) CD spectrum of CoV-2-p5 (dashed line; in absence of TFE, straight line; in presence of 25% TFE)

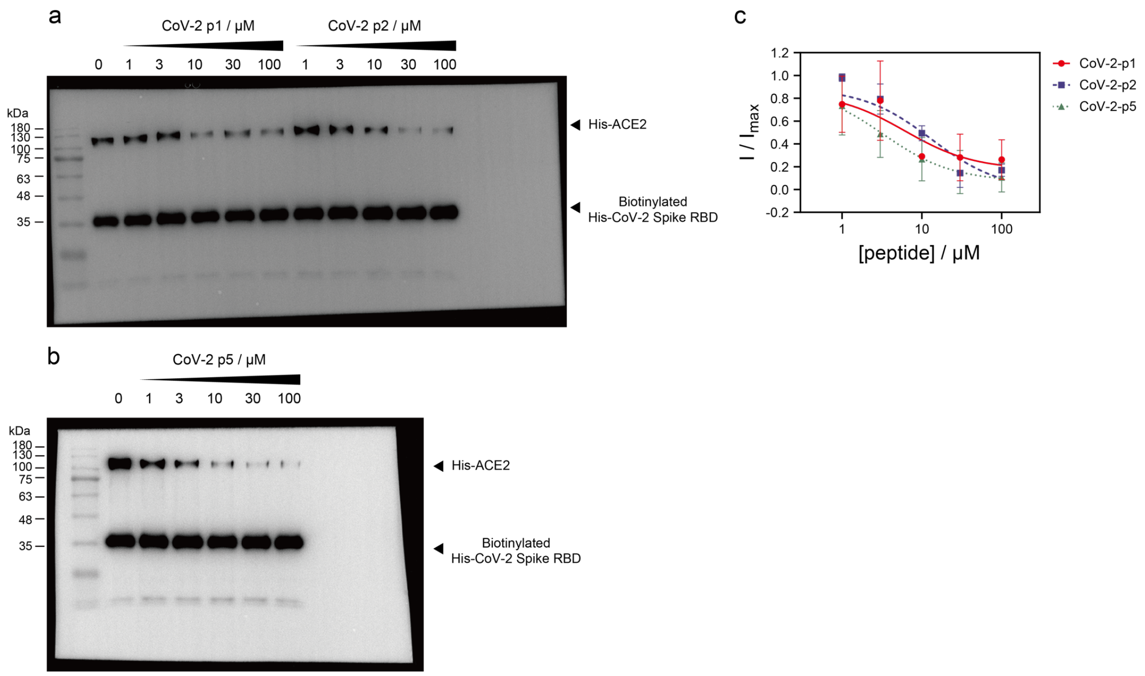

Fig. S20 Pull-down competition assay results of CoV-2-p1, p2, and p5. Uncropped western blotting images of pull-down competitive assay (a) CoV-2-p1 and p2 and (b) CoV-2-p5. (c) Concentration dependent decrease of bound ACE2. Normalized bond intensities of ACE2 were plotted (n = 3, error bar means SD).

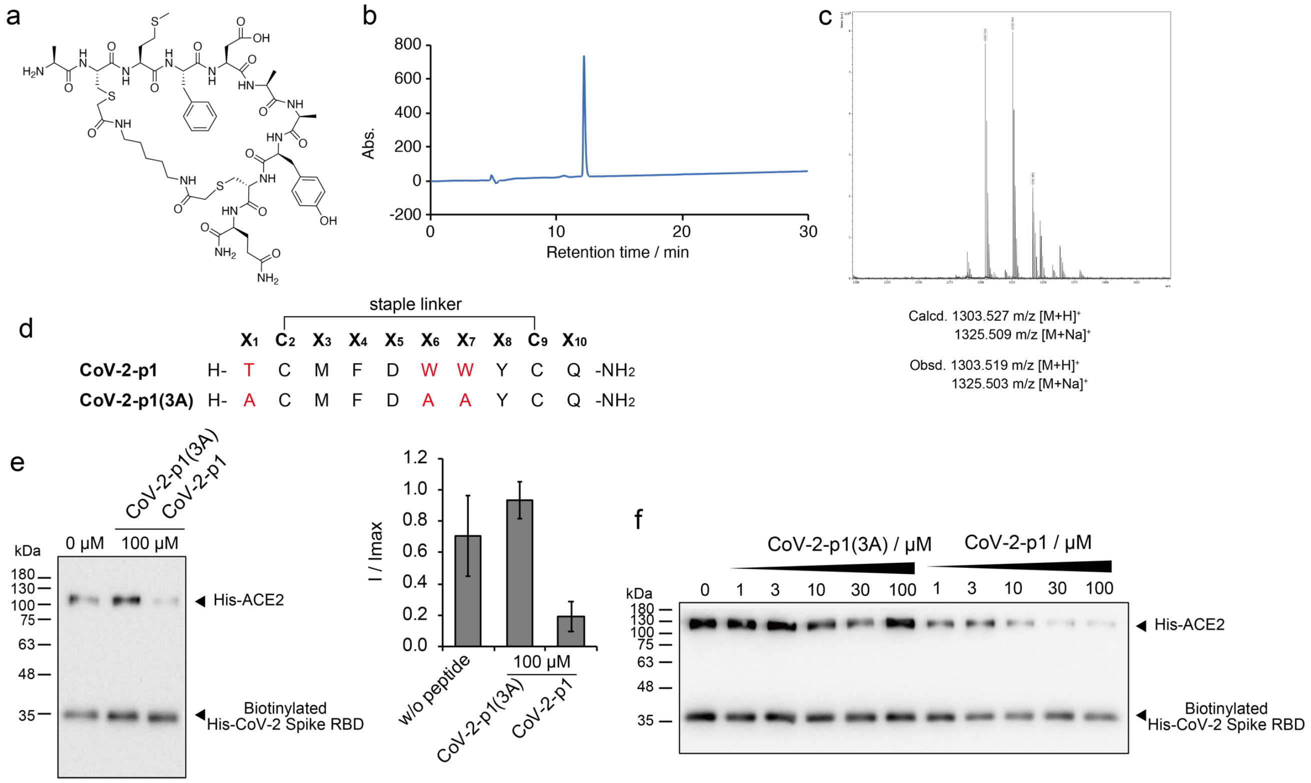

Fig. S21 Pull-down competition assay results of CoV-2-p1(3A). (a) Chemical structure of CoV-2-p1(3A). (b) Analytical HPLC for purity check. (c) MALDI-TOF MS results. (d) A list of peptides tested in pull-down competition assay. (e) Pull-down competition assay results of CoV-2-p1(3A) and p1. To the immobilised biotinylated CoV-2 Spike RBD, recombinant ACE2 (15 μg/mL) and peptides (100 μM) were added. The resins were washed with HBS-T one hour after incubation, followed by the elution, SDS-PAGE and western blotting using an HRP-conjugated His-tag monoclonal antibody (proteintech, HRP-7705, 1/1,000 dilution). (n = 3, error bar means SD) (f) Dose dependency of CoV-2-p1(3A) and p1 in the pull-down competition assay.

Table S1. Summary of NGS results and the processing (Amp-R1, R2, and R3)

Sample	Replication	Pair-end	Raw fastq	Trimmed fastq	DNA match fastq	Q-filtered fastq	Pair matched fastq	Merged fasta	Unique peptide
Amp R1	1	forward	211,916	193,497	189,443	174,086	165,541	389,893	371,325
		reverse	211,916	195,303	191,318	176,661			
	2	forward	112,426	103,816	101,375	94,467	89,790		
		reverse	112,426	103,093	100,965	94,622			
	3	forward	167,963	155,280	151,562	140,162	134,562		
		reverse	167,963	155,062	151,905	142,800			
Amp R3	1	forward	120,589	112,303	109,980	105,417	101,766	414,111	312,278
		reverse	120,589	111,551	109,205	104,894			
	2	forward	207,618	189,217	182,980	166,982	159,699		
		reverse	207,618	193,091	189,299	172,856			
	3	forward	197,799	180,262	174,352	159,731	152,646		
		reverse	197,799	183,919	180,261	164,732			
Amp R5	1	forward	167,746	157,557	154,176	140,618	125,178	125,178	46,728
		reverse	167,746	148,063	142,438	131,519			

Table S2. Summary of NGS results and the processing (HDM2-R1, R2, and R3)

Sample	Pair-end	Raw fastq	Trimmed fastq	DNA match fastq	Q-filtered fastq	Pair matched fastq	Unique peptide
HDM2-R1	forward	204,285	184,833	180,207	165,880	156,091	104,779
	reverse	204,285	185,698	181,096	165,755		
HDM2-R2	forward	197,835	181,480	177,499	163,408	154,864	51,673
	reverse	197,835	182,876	178,956	165,076		
HDM2-R3	forward	147,934	137,663	134,393	124,248	119,799	27,253
	reverse	147,934	137,533	134,711	126,994		

Table S3. Summary of pattern enrichment analysis

Input		Output					
Sample	Total sequences	Found patterns	Coverage %	Theoritical patterns	Median counts	Average counts	Standard deviation
Amp-R3	414,111	402,837	89.9	448,000	14	51.8	125.1
HDM2-R3	119,799	288,379	64.4	448,000	3	15.0	46.1
RBD C5 R3	132,024	132,737	29.6	448,000	0	17.1	163.8

Table S4. Summary of NGS results and the processing (SARS-CoV-2 Spike RBD-R1, R2, and R3)

Sample	Pair-end	Raw fastq	Trimmed fastq	DNA match fastq	Q-filtered fastq	Pair matched fastq	Unique peptide
SARS-CoV-2 Spike RBD-R1	forward	199,744	185,691	182,056	170,362	161,280	61,785
	reverse	199,744	184,410	180,894	165,868		
SARS-CoV-2 Spike RBD-R2	forward	184,659	169,880	166,418	154,876	146,786	8,920
	reverse	184,659	168,870	165,595	151,287		
SARS-CoV-2 Spike RBD-R3	forward	162,178	150,790	147,666	138,120	132,024	5,504
	reverse	162,178	150,055	147,051	136,022		

Table S5. A list of synthesised peptides against HDM2

			staple linker											
		X_1	C_2	X_3	X_4	X_5	X_6	X_7	X_8	C_9	X_{10}		**Helical content* (25% TFE)**	**IC50 / μM†**
**HDM2-p13**	H-	A	C	F	D	E	Y	W	Q	C	L	$-NH_2$	42.8%	4.8 ± 0.6
**HDM2-opt**	H-	P	C	F	Y	D	Y	W	Q	C	L	$-NH_2$	26.0%	6.9 ± 1.9

*Helical contents were calculated by BeStSel web server[5].

†The competitive inhibition of the interaction between HDM2 (100 nM) and FAM-ATSP-3848 (10 nM) was tested for HDM2-p13 and opt by fluorescence polarization assay.

Table S6. A list of synthesised peptides against SARS-CoV-2 Spike RBD

			staple linker											
		X_1	C_2	X_3	X_4	X_5	X_6	X_7	X_8	C_9	X_{10}		**Helical content* (25% TFE)**	**IC50 / μM†**
**CoV-2-p1**	H-	T	C	M	F	D	W	W	Y	C	Q	$-NH_2$	39.1%	7.8 ± 8.2
**CoV-2-p2**	H-	T	C	L	S	S	W	F	E	C	F	$-NH_2$	44.8%	12.9 ± 4.9
**CoV-2-p5**	H-	T	C	W	G	F	D	Y	L	C	W	$-NH_2$	11.6%	3.8 ± 3.3

*Helical contents were calculated by BeStSel web server[5].

†The competitive pull-down assay of CoV-2-p1, p2, and p5 was conducted against the interaction between immobilized SARS-CoV-2 spike RBD and soluble ACE2.

Reference

1 I. Rentero Rebollo and C. Heinis, *Methods*, 2013, **60**, 46–54.

2 T. Ananuchatkul, H. Tsutsumi, T. Miki and H. Mihara, *Bioorganic Med. Chem. Lett.*, 2020, **30**, 127605.

3 I. R. Rebollo, M. Sabisz, V. Baeriswyl and C. Heinis, *Nucleic Acids Res.*, 2014, **42**, e169.

4 W. L. Matochko, K. Chu, B. Jin, S. W. Lee, G. M. Whitesides and R. Derda, *Methods*, 2012, **58**, 47–55.

5 A. Micsonai, F. Wien, L. Kernya, Y. H. Lee, Y. Goto, M. Réfrégiers and J. Kardos, *Proc. Natl. Acad. Sci. U. S. A.*, 2015, **112**, E3095–E3103.

6 T. L. Bailey, *Bioinformatics*, 2021, **37**, 2834–2840.

7 C. Li, M. Pazgier, C. Li, W. Yuan, M. Liu, G. Wei, W. Y. Lu and W. Lu, *J. Mol. Biol.*, 2010, **398**, 200–213.

8 S. Baek, P. S. Kutchukian, G. L. Verdine, R. Huber, T. A. Holak, K. W. Lee and G. M. Popowicz, *J. Am. Chem. Soc.*, 2012, **134**, 103–106.

9 Y. S. Chang, B. Graves, V. Guerlavais, C. Tovar, K. Packman, K. H. To, K. A. Olson, K. Kesavan, P. Gangurde, A. Mukherjee, T. Baker, K. Darlak, C. Elkin, Z. Filipovic, F. Z. Qureshi, H. Cai, P. Berry, E. Feyfant, X. E. Shi, J. Horstick, D. A. Annis, A. M. Manning, N. Fotouhi, H. Nash, L. T. Vassilev and T. K. Sawyer, *Proc. Natl. Acad. Sci. U. S. A.*, 2013, **110**, E3445–E3454.
